# Structure‐Guided Engineering of a Versatile Urethanase Improves Its Polyurethane Depolymerization Activity

**DOI:** 10.1002/advs.202416019

**Published:** 2025-02-07

**Authors:** Zhishuai Li, Xu Han, Lin Cong, Parinita Singh, Pedro Paiva, Yannick Branson, Wenshuo Li, Yangyang Chen, Da'san M. M. Jaradat, Frank Lennartz, Thomas Bayer, Louis Schmidt, Ulrike Garscha, Song You, Pedro Alexandrino Fernandes, Maria João Ramos, Uwe T. Bornscheuer, Gert Weber, Ren Wei, Weidong Liu

**Affiliations:** ^1^ Key Laboratory of Engineering Biology for Low‐carbon Manufacturing National Engineering Research Center for Industrial Enzymes National Technology Innovation Center of Synthetic Biology Tianjin Institute of Industrial Biotechnology Chinese Academy of Sciences 32 West Seventh Avenue, Tianjin Airport Economic Area Tianjin 300308 China; ^2^ University of Chinese Academy of Sciences 19A Yuquan Road Beijing 100049 China; ^3^ School of Life Sciences and Biopharmaceutical Sciences Shenyang Pharmaceutical University 103 Wenhua Road Shenhe District Shenyang Liaoning 110016 China; ^4^ Helmholtz‐Zentrum Berlin für Materialien und Energie Albert‐Einstein‐Str. 15 D‐12489 Berlin Germany; ^5^ LAQV@REQUIMTE Departamento de Química e Bioquímica Faculdade de Ciências da Universidade do Porto Rua do Campo Alegre Porto 4169‐007 Portugal; ^6^ Department of Biotechnology and Enzyme Catalysis Institute of Biochemistry University of Greifswald Felix‐Hausdorff‐Str. 4 17487 Greifswald Germany; ^7^ Department of Chemistry Faculty of Science Al‐Balqa Applied University P.O. Box 206 Al‐Salt 19117 Jordan; ^8^ Department of Pharmaceutical and Medicinal Chemistry Institute of Pharmacy University of Greifswald Friedrich‐Ludwig‐Jahn‐Str. 17 17489 Greifswald Germany

**Keywords:** crystal structures, enzyme engineering, molecular dynamics simulations, plastic degradation, polyurethane, urethanase

## Abstract

Polyurethane (PUR), the fifth most prevalent synthetic polymer, substantially contributes to the global plastic waste problem. Biotechnology‐based recycling methods have recently emerged as innovative solutions to plastic waste disposal and sparked interest among scientific communities and industrial stakeholders in discovering and designing highly active plastic‐degrading enzymes. Here, the ligand‐free crystal structure of UMG‐SP2, a metagenome‐derived urethanase with depolymerization activities, at 2.59 Å resolution, as well as its (co‐)structures bound to a suicide hydrolase inhibitor and a short‐chain carbamate substrate at 2.16 and 2.40 Å resolutions, respectively, is reported. Structural analysis and molecular dynamics simulations reveal that the flexible loop L3 consisting of residues 219–226 is crucial for regulating the hydrolytic activity of UMG‐SP2. The semi‐rational redesign of UMG‐SP2 reveals superior variants, A141G and Q399A, exhibiting over 30.7‐ and 7.4‐fold increased activities on polyester‐PUR and a methylene diamine derivative of PUR, respectively, compared to the wild‐type enzyme. These findings advance the understanding of the structure–function relationship of PUR‐hydrolyzing enzymes, which hold great promise for developing effective industrial PUR recycling processes and mitigating the environmental footprint of plastic waste.

## Graphical abstract

The crystal structures of UMG‐SP2, a metagenome‐derived urethanase that degrades polyurethane, have been solved at high resolution in both ligand‐free and ligand‐bound forms with an amidase inhibitor or a small carbamate. Structure‐guided engineering of UMG‐SP2 improves the understanding of polyurethane degradation mechanisms and generates enzyme variants with over 30.7‐fold higher depolymerization activities, promising bio‐recycling of waste polyurethane.

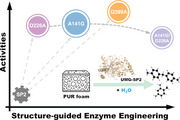

## 1 Introduction

The global plastic waste problem has reached a critical point due to its omnipresence in landfills and natural habitats, representing substantial ecological risks.^[^ ^1^ ^]^ Despite the growing plastics production from recycled or bio‐based feedstocks, the vast majority of virgin plastics are still derived from fossil fuels.^[^ ^2^ ^]^ As a result, petrochemical‐based waste plastics continue to dominate the solid waste management system, necessitating environment‐friendly disposal and recycling methods.^[^ ^3^ ^]^ Recently, innovative bio‐based plastic recycling and upcycling processes have emerged that use engineered enzymes and microbes.^[^ ^4^ ^]^ One prominent example is the industrial‐scale enzymatic recycling of polyethylene terephthalate (PET), which is widely used in single‐use bottles and clothing, as a result of scientific advances in polyester hydrolases over the last two decades.^[^ ^5^ ^]^ Following this success, scientific communities have continued to seek biocatalytic solutions for other waste plastics with hydrolysable backbones, such as polyurethane (PUR),^[^ ^6^ ^]^ the fifth most commonly produced plastic type.^[^ ^2^ ^]^

While PUR products (e.g., foams, elastomers, coatings, and adhesives^[^ ^7^ ^]^) have usually a longer lifespan than PET or other commonly used plastics in disposable items, their complex chemical compositions and eventually cross‐linked polymer chains (i.e., as thermosets) make enzymatic recycling and biodegradation under natural conditions extremely challenging.^[^ ^8^ ^]^ The different types of polyols, di‐isocyanates, and chain extenders used for PUR synthesis determine polymer properties associated with biodegradability.^[^ ^7^ ^]^ For instance, the ester bonds in the polyol soft segments, which often exist as disordered amorphous microstructures, are more prone to enzymatic attack by ester hydrolases, whereas the urethane bonds in the crystalline hard segments, usually additionally reinforced by hydrogen bonds between polymer chains, are much less susceptible to biological degradation.^[^ ^6^ ^]^ Therefore, most PUR‐hydrolyzing enzymes reported so far are polyester hydrolases, which cannot act on the chemically more stable carbamate bond in (poly)urethanes.^[^ ^6^, ^9^ ^]^ While there have been several reports on the biological decomposition of polyether‐based PUR,^[^ ^10^ ^]^ which contains exclusively hydrolysable carbamate bonds, only a few members of the amidase superfamily (EC 3.5.1) have been reported for their ability to hydrolyze urethane bonds in PUR or related low‐molecular‐weight carbamates.^[^ ^11^ ^]^ Out of these few specific examples, the metagenome‐derived urethanases (UMG‐SP1, UMG‐SP2, and UMG‐SP3), discovered by us,^[^ ^11^ ^]^ have sparked significant interest in scientific communities and industrial stakeholders.^[^ ^12^ ^]^ We have recently solved the crystal structures of UMG‐SP1 and UMG‐SP3 by X‐ray crystallography and performed structure‐based protein engineering to evaluate and improve their hydrolytic activity on small‐molecule substrates, as well as to depolymerize PUR and polyamides (PA, e.g., nylon 6) materials.^[^ ^13^ ^]^ Thus, all these urethanases are believed to possess significant potential for the future advancement of bio‐recycling processes for waste PUR and PA.

**Figure 1:**
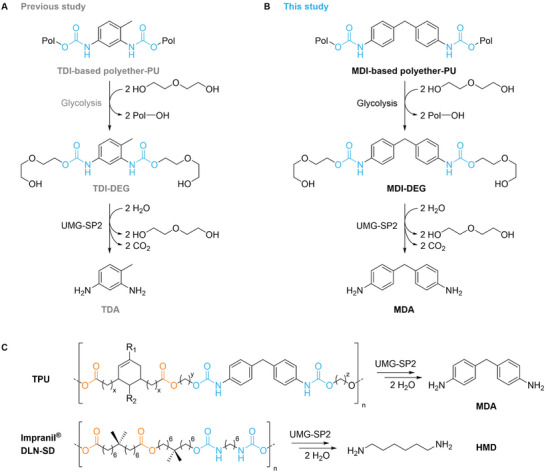
Biocatalytic applications of UMG‐SP2. A) In our previous study, toluene diisocyanate (TDI)‐based polyether‐PUR waste was chemically degraded through glycolysis at 200 °C in the presence of diethylene glycol (DEG = C_4_H_10_O_3_), yielding the dicarbamate TDI‐DEG.^[^ ^11c^ ^]^ Urethane groups (blue) were hydrolyzed by UMG‐SP2, forming 2,4‐diaminotoluene (TDA) and 2,6‐diaminotoluene (not shown here for clarity), CO_2_, and DEG. B) In this study, a methylene diphenyl diisocyanate (MDI)‐based polyether‐PUR was degraded by glycolysis accordingly. The produced MDI‐DEG was hydrolyzed by UMG‐SP2, yielding the diamine 4,4′‐methylenedianiline (MDA). C) UMG‐SP2 was also utilized to degrade a model thermoplastic polyester‐PUR (TPU; R_1_ and R_2_ = aliphatic side chains) and Impranil DLN‐SD, a commercial anionic aliphatic polyester‐PUR dispersion,^[^ ^14^ ^]^ producing the monomers MDA and hexamethylenediamine (HMD), respectively. Ester bonds are shown in orange. Other degradation products are not shown for clarity. The lengths of the alkyl chains (x, y, and z) are excluded owing to a nondisclosure agreement with Soprema.

In this study, we report the three‐dimensional structure of UMG‐SP2, which shares 52.4% sequence identity with UMG‐SP1 but has higher or comparable hydrolytic activity on PA and urethane oligomers.^[^ ^11^, ^12^ ^]^ Beyond the previously reported substrate‐free structure of UMG‐SP1,^[^ ^13a^ ^]^ we solved co‐structures of UMG‐SP2 bound with a suicide inhibitor (SP2^PMS^) or a short‐chain urethane substrate using an active site mutant (SP2^S190A^). The ligand‐bound UMG‐SP2 structures, combined with our molecular dynamics (MD) simulations, provide significant insights into the architecture of the active site and the mechanisms involved in substrate binding. In addition, we performed structure‐guided mutagenesis of UMG‐SP2 to generate variants with improved activity on both short‐chain substrates like diamine 4,4′‐methylenedianiline (MDI‐DEG) and various PUR substrates (**Figure** **1**). Our findings shed light on the structure–function relationship of urethane‐hydrolyzing enzymes, which could pave the way for bio‐based industrial PUR recycling processes.

## 2 Results and Discussion

### 2.1 UMG‐SP2 Structures in Free and Ligand‐Bound Forms

**Figure 2:**
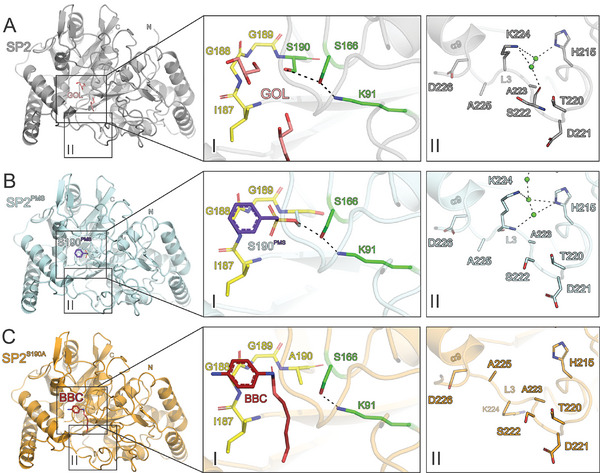
UMG‐SP2 displays an amidase fold and elicits a unique conformational switch upon substrate binding. A) The overall structure of SP2 colored in grey with glycerol (GOL) molecules in the active site shown in salmon. B) The overall structure of SP2^PMS^ colored in light blue with the covalently bound PMS shown in purple. C) The overall structure of SP2^S190A^ colored in light orange with the substrate BCC shown in firebrick red. The left inset (I) shows a close‐up of the active site with the oxyanion hole colored in yellow and the catalytic triad residues in green. The right inset (II) shows the conformational switch with K224 pointing into (A, B) or out of (C) the active site cavity for each structure. Dashed lines indicate hydrogen bonds. Interacting residues are shown as sticks and colored by atom type: carbon as given for the respective molecule, nitrogen in blue, oxygen in red and sulfur in yellow. Water oxygens are shown as green spheres. The original residue numbering under GenBank accession number OP972510 (without the N‐terminal expression tag and linker) was adjusted to make the residue order more consistent with the crystal structures uploaded to the PDB database (Table S1, Supporting Information).

As shown in **Figure** **2**, the crystal structures of wild‐type (WT) UMG‐SP2 (GenBank Accession No. OP972510) were solved in a substrate‐free state (SP2, also used as an interchangeable designation for WT UMG‐SP2 for simplification, 2.59 Å resolution) and a complex with the phenylmethylsulfonyl moiety of the suicide inhibitor PMSF covalently bound to the active site serine 190 (SP2^PMS^, 2.16 Å resolution). In addition, a SP2 variant with the catalytic triad serine mutated to alanine (S190A) was created and used to solve the complex structure (SP2^S190A^, 2.40 Å resolution) with the model substrate 4‐hydroxybutyl (4‐(4‐aminobenzyl)phenyl) carbamate (BBC) containing a urethane bond. The structure of SP2^PMS^ was solved by molecular replacement (MR), employing the structural coordinates of a structural model of UMG‐SP2 created by AlphaFold2 (AF2).^[^ ^11^, ^15^ ^]^ The structures of SP2^S190A^ and SP2 were solved by MR based on the refined structure of SP2^PMS^ omitting the covalently modified S190. All structures were iteratively refined to low R/R_free_ factors with good stereochemistry,^[^ ^15^ ^]^ yielding electron density of high quality for all residues and substrates in both structures apart from the flexible N‐ and C‐termini (Table S1 and Figures S1A–C and S2A, Supporting Information).

#### 2.1.1 Comparison of UMG‐SP2 Structures in Various Substrate‐Binding Modes

**Figure 3:**
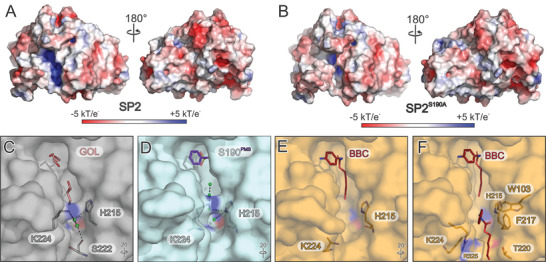
Electrostatic surface potentials and accessibility of the active site channel in the SP2 structures. A,B) Electrostatic surface potentials as indicated by the color scale bar (bottom), obtained by APBS version 3.4.1^[^ ^17^ ^]^ and plotted on the surface of SP2 (A) and SP2^S190A^ bound to BBC (B, ligand not shown). C–F) Comparison of the active site cavities of SP2, SP2^PMS^, and SP2^S190A^ bound to BBC. The structural superimposition compares the surface of the active site cavities of SP2 (C), SP2^PMS^(D), SP2^S190A^ bound to BBC (E), and the second molecule of SP2^S190A^ in the asymmetric unit where an additional BBC substrate binding was found at the edge of the active site occupying binding site 2 (F). Glycerol (GOL), PMS, and BBC, as well as K224 and H215 and other residues maintaining contact with the second BBC substrate (F) are shown as sticks. Colors and molecule representations are the same as in Figure 2, with rotational symbols relative to Figure 2A. Water molecules are shown as green spheres, hydrogen bonds as dashed lines.

UMG‐SP2 belongs to the amidase signature protein superfamily (AS family), which is characterized by a highly conserved GGSS(S/G)GS motif and can be found in bacteria, archaea, and eukaryotes.^[^ ^16^ ^]^ SP2 shares the domain architecture of amidases, with an α/β‐fold containing 12 prominent α‐helices, 12 β‐strands, and five larger loops. Due to the hydrophobic nature of the entire active site cavity, two glycerol molecules from the cryoprotectant solution were found bound to the active site in the substrate‐free structure, one of which contacted the backbone of the oxyanion hole residues (Figure 2A, left inset, **Figure** **3**C, and S1A, Supporting Information). The remainder of the suicide inhibitor PMSF, PMS, bound to the WT SP2 clearly illustrates the topology of the active site architecture, with the amides of residues I187–S190 forming an oxyanion hole and K91, *cis*S166, and S190 representing the catalytic triad (Figure 2B, left inset, Figure S1B, Supporting Information). Likewise, when bound to the primary active site cavity of the inactive mutant SP2^S190A^, the phenyl moiety of the substrate BBC adopts a similar pose as PMS, with the scissile urethane bond in the vicinity of A190 (Figure 2C, left inset, Figure S1C,D, Supporting Information). The aliphatic 4‐hydroxybutyl moiety of BBC crosses the oxyanion hole and enters a secondary cavity separate from the active site. This secondary cavity (Figure 2, right insets) may facilitate the accommodation of substrates larger than BBC, such as the dicarbamate TDI‐DEG (Figure 1A).^[^ ^11c^ ^]^

The overall fold of all structures is highly similar, with root mean square deviations (RMSD) of Cα atoms between SP2 and SP2^PMS^ or SP2^S190A^ of 0.2 and 0.25 Å, respectively. Likewise, there is a high similarity to the AF2 model of SP2^AF2^ with Cα RMSD of 0.71, 0.6, and 0.65 Å to SP2, SP2^PMS^, and SP2^S190A^, respectively (Figure S1F, Supporting Information). Only one region near the active site of the substrate‐bound SP2^S190A^, belonging to the loop L3 (residues 219–226, Figure S2A, Supporting Information, corresponding to the loop L3 also found in UMG‐SP1^[^ ^13^ ^]^), has a different conformation than the other structures (Figure 2A–C) and the computational model SP2^AF2^ (Figure S1F, Supporting Information). In SP2 and SP2^PMS^, K224 (part of L3 at the perimeter of the secondary active site cavity) interacts with several water molecules and H215 (as well as S222 in the SP2 structure). These interactions consolidate K224 pointing toward the substrate binding cavity and blocking the secondary active site sterically (Figure 2A–C, right insets). Upon binding of BBC, K224, and concomitantly the flanking residues A223 and A225 perform a backbone flip that leads to a release of the active site steric blockage and the K224 side chain now points into the solvent as seen in the SP2^S190A^ structure complexed with BBC (Figure 2C, right insert). Due to the smaller side chains of the two flanking alanine residues, it is only K224 that blocks the secondary active site cavity and releases this steric hindrance by the aforementioned backbone flip with no major changes in the proximity (Figure 2A–C, right insets, Figure 3C–F).

Interestingly, we discovered a second substrate binding site that is only found in the second molecule of the asymmetric unit of the BBC‐bound SP2^S190A^ complex structure (Figure 3F, Figure S1E, Supporting Information). In a previous study, we described similar multiple substrate binding modes with PES‐H1 and PES‐H2, two metagenome‐derived PET hydrolases.^[^ ^17^ ^]^ Thus, it can be deduced that polymer‐degrading enzymes could potentially gain advantages from possessing supplementary substrate binding sites where catalysis‐unrelated binding is fostered. This observation may also mirror the environment at the surface of larger solid substrates where several scissile bonds are located adjacent to each other. This second substrate binding site in UMG‐SP2, formed by the residues W103, F217, T220, and R325, coincides with the secondary substrate cavity regulated by K224 and may serve as an entry point for the substrate. As a result, K224 may also control substrate release through the secondary active site cavity. We hypothesize that K224 is part of a substrate‐gating mechanism, also observed in other enzymes^[^ ^18^ ^]^ but not yet described for members of the AS family. L3 may regulate substrate entry (or release) by altering its backbone conformation, causing the side chain of K224 to point into the active site for substrate release or into the solvent for substrate binding.^[^ ^13^ ^]^

The surface of SP2 is largely negatively charged, but positive charge overweighs the substrate‐free structure in the primary and secondary active site cavities, in contrast to the BBC‐bound SP2^S190A^. This charge difference is also the result of the aforementioned structural switch that distinguishes SP2 from the substrate‐bound complex structure of SP2^S190A^ (Figure 3A,B). Based on three different SP2–substrate interactions (PMS‐bound, one BBC, and two BBC in the active site), a regulation mechanism elicited by K224 is corroborated. Due to the steric blockage of the active site when empty (Figure 3C) or partially occupied by PMS (Figure 3D), substrate accessibility (in particular for larger molecules) is limited, in contrast to the complex structures with one (Figure 3E) or two BBC molecules bound (Figure 3F). Given that all structures are present in the identical crystal lattice, specifically the same space group, and arranged in an isomorphous manner, we can question the possibility of the crystal lattice or crystal packing‐related influences on the observed conformational backbone flip.

#### 2.1.2 UMG‐SP2 Structures Compared to Other Related Enzymes

A search for structural homologs to UMG‐SP2 employing the FoldSeek^[^ ^19^ ^]^ and DALI^[^ ^20^ ^]^ servers yielded several structurally related enzymes of the AS family from the PDB database. We selected the enzymes with the closest structural similarities, focusing on substrate‐bound structures, for further investigation and comparison. In a structure‐based sequence alignment employing these enzymes, as well as computational models of UMG‐SP1 (SP1^AF2^) and UMG‐SP3 (SP3^AF2^), several hallmarks and a high degree of secondary structure conservation of this enzyme family (like the conserved GGSS(S/G)GS‐motif or the K‐*cis*S‐S catalytic triad) are evident (Figure 5, Figure S2A, Supporting Information).^[^ ^16^ ^]^ SP2 and the related structures discovered in the search share a high degree of structural and sequence similarity. The candidate enzymes from the AS family had relatively low RMSD to our reference SP2 structure, for example, nylonase A^[^ ^21^ ^]^ (NylA, PDB‐ID: 3A2P, Cα RMSD 1.6 Å, 374 residues aligned, 31% sequence identities), hydrazidase^[^ ^22^ ^]^ (HZD, PDB‐ID: 5H6S, Cα RMSD 1.8 Å, 373 residues aligned, 29% sequence identities), 1‐carboxybioret amidase^[^ ^23^ ^]^ (AtzE, PDB‐ID: 6C6G, Cα RMSD 1.7 Å, 385 residues aligned, 35% sequence identities), and the amidase ClbL^[^ ^24^ ^]^ (ClbL, PDB‐ID: 8ES6, Cα RMSD 1.4 Å, 389 residues aligned, 35% sequence identities). Interestingly, a dimer of the HZD asymmetric unit superimposes on the two molecules of the SP2 asymmetric unit with an RMSD of 3.3 Å, indicating a highly similar dimer formation, albeit with some variance due to different positioning of one of the molecules, that is unlikely a coincidence (Figure S3A, Supporting Information). L3 of HZD has an R208 residue that points away from the secondary cavity of the active site in both structures of HZD (PDB‐ID: 5H6S, 5H6T), but it is similar to K224 in SP2. Like K224 in SP2, R208 in HZD is flanked by two relatively flexible amino acid residues, G207 and S209. Taken together, we cannot rule out the possibility of functional relevance for SP2 dimerization in the asymmetric unit cells of our crystals.

### 2.2 Structure‐Based Engineering of UMG‐SP2

To study the structure–function relationship and to improve the hydrolytic activity of UMG‐SP2, amino acids within 5 Å of the BBC ligand, as well as a few more distant residues (e.g., A141 and Q399) were chosen for site‐specific mutagenesis. One mutagenesis strategy involves substituting bulky residues with either alanine or glycine (only if the wild‐type residue is alanine) to anticipate a larger substrate binding pocket, while other residue substitutions were designed for a more fundamental mechanistic comprehension. Certain expectedly inactive variants related to the catalytic triad, including K91A, S167A (next to the *cis*S166), and S190A, were also generated for crystallographic studies to soak substrate‐like ligands. K224 in the flexible loop L3 (Figure S2A, Supporting Information) was substituted by various residues through semi‐site‐saturated mutagenesis, while the neighboring D226 was replaced with residues found in homologous enzymes (e.g., UMG‐SP1).^[^ ^11^, ^13^ ^]^ In addition, promising single mutants were combined, yielding double mutants (e.g., A141G/D226A).

#### 2.2.1 Comparative Analysis of UMG‐SP2 Mutant Activity on Various Substrates

Enzyme variants, obtained through site‐directed mutagenesis, were expressed recombinantly in *Escherichia coli* BL21(DE3) and purified before activity assessment using different substrates containing urethane bonds. Similar to our previous approach,^[^ ^11^ ^]^ we obtained a small‐molecule substrate through glycolysis of post‐consumer polyether‐based PUR waste (Figure 1B). This process yielded MDI‐DEG, which consists of a central MDA molecule connected to two DEG molecules by two carbamate bonds. MDI‐DEG hydrolysis produces an intermediate in which only one carbamate bond is cleaved. The intermediate could be identified using LC‐MS (Figure S4A, Supporting Information), but could not be quantified due to the lack of a commercially available standard compound. After complete enzymatic hydrolysis, MDA is released, which can be accurately quantified, using a calibration curve (Figure S4B, Supporting Information) recorded with commercial MDA.

**Figure 4:**
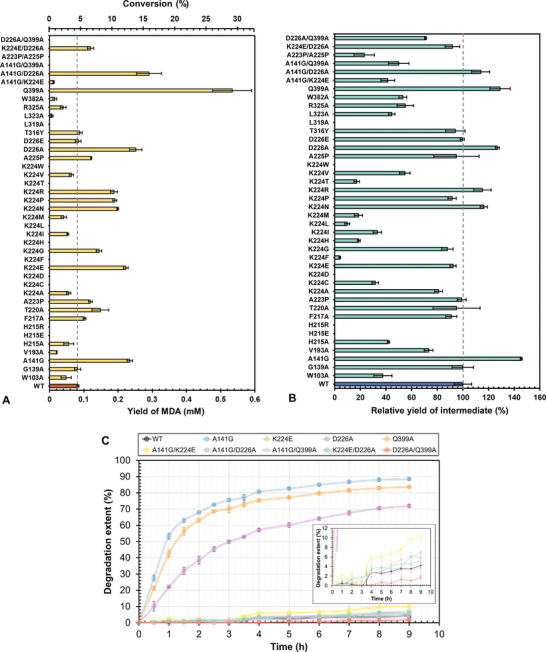
Enzymatic degradation of MDI‐DEG and polyester‐PUR particles catalyzed by UMG‐SP2 and its variants. A) MDA yield (mm) from the hydrolysis of MDI‐DEG after 1 h reaction according to LC‐MS measurements. The percentage conversion from MDI‐DEG to MDA was calculated using an estimated total MDI‐DEG concentration of 1.837 mm in each reaction vial, as described in the Experimental Section. B) The corresponding percentage yields of the intermediate, relative to those determined with the WT enzyme. C) Time courses of percentage degradation extents of a polyester‐PUR suspension (0.4%, w/v, Impranil DLN‐SD) over 9 h. The turbidity decrease was measured at 400 nm as a function of time. After subtracting the turbidity determined with the blank sample (without enzyme), the percentage degradation extents were calculated against turbidity determined before the addition of enzymes (*t* = 0 h). Three single variants—D226A (lavender), Q399A (light orange), and A141G (light blue)—markedly outperformed the WT enzyme (black), with the degradation time courses of it and other less active variants clearly illustrated in an enlarged inset in (C). Results are presented as mean values ± standard deviation (SD) from three independent experiments.

**Figure** **4**A shows the yield of MDA after the enzymatic hydrolysis of MDI‐DEG for 1 h using WT SP2 and its variants. Among the single variants, Q399A, D226A, A141G, K224E, K224N, K224P, K224R, T220A, K224G, A225P, A223P, and F217A, arranged in descending order of MDA yield, showed improved hydrolytic activity against MDI‐DEG. The maximum MDA yield of 0.534 ± 0.057 × 10^−3^ m obtained with Q399A is 7.4 times greater than that produced by the WT enzyme. A similar MDA yield level was obtained with G139A in comparison to the WT enzyme, whereas other mutants exhibited considerably lower activities. No MDA release was detected with H215E, H215R, K224C, K224D, K224F, K224H, K224L, K224T, K224 W, and L319A. Figure 4B illustrates the relative yields of the intermediate following the hydrolysis of only one carbamate bond in MDI‐DEG (Figure 1B) before the second hydrolysis to yield the final product MDA. The data suggest that A141G, Q399A, D226A, K224N, and K224R are superior SP2 variants for this single hydrolysis step. Several inactive variants at position K224 (K224C, K224F, K224H, K224L, and K224T) indicated by the MDA yields (Figure 4A) showed measurable levels of intermediate release, suggesting their limitations in catalyzing the hydrolysis of the second carbamate in MDI‐DEG with a symmetric conformation. In contrast, H215E, H215R, K224D, K224 W, and L319A have been proven to be completely inactive variants for MDI‐DEG hydrolysis. Given the aforementioned activity ranking and the significance of MDA yield requiring two hydrolytic reactions, we generated five double mutants by combining Q399A, D226A, A141G, and K224E, and then characterized them in MDI‐DEG hydrolysis (Figure 4). Surprisingly, only the double mutant A141G/D226A showed higher activity than the WT enzyme in terms of both MDA and intermediate release, with values significantly lower than those obtained with Q399A, the superior single variant. In addition, we combined A223P and A225P for a more comprehensive understanding of their structural roles as adjacent residues to K224. The resultant double mutant exhibited significantly reduced intermediate yield relative to the WT enzyme and did not produce MDA, similar to A141G/Q399A and D226A/Q399A, despite the latter two double mutants containing the advantageous residue substitution of Q399A.

Next, we evaluated the capabilities of selected single and double variants in depolymerizing Impranil DLN‐SD, a commercially available waterborne polyester‐PUR suspension (Figure 1C). A suspension containing 0.4% (w/v) polymer material was used to monitor the turbidimetric decrease at 400 nm over 9 h at 30 °C after adding individual enzyme variants (Figure 4C) as performed previously.^[^ ^9a^ ^]^ WT SP2 has been shown to retain 44% of its initial activity following 12 h incubation at 29.4 °C.^[11c]^ Therefore, we assessed the melting temperatures (*T*_m_) of different enzyme variants (Table S2, Supporting Information) using differential scanning fluorimetry (DSF) to eliminate the possible impact of their altered thermostability on the Impranil hydrolysis over 9 h. The WT enzyme demonstrated a *T*_m_ of 44.0 ± 0.1 °C, while the SP2 variants evaluated (Figure 4C) showed no significant stabilization or destabilization due to mutagenesis, exhibiting a *T*_m_ range of 42.1–48.3 °C. Consequently, their varying performance in PUR depolymerization was attributed exclusively to their modified hydrolytic activity. A141G, Q399A, and D226A resulted in over 50%, 40%, and 20% PUR degradation within 1 h, respectively, while no notable degradation was observed with other enzyme variants until 4 h (Figure 4C). The degradation curves of the three superior variants begin to plateau after the first hour of reaction, whereas other enzyme variants exhibit an apparent linear increase in degradation performance from 4 to 9 h of the hydrolysis reaction. After 4 h, A141G, Q399A, and D226A exhibited degradation performances that were 30.7, 28.7, and 21.7 times greater than the WT SP2, respectively. Among the tested double mutants, only A141G/K224E clearly outperformed the WT enzyme in PUR depolymerization, but it was still far less active than the three superior single mutants. Other variants, including A141G/D226A, which had higher hydrolytic activity on MDI‐DEG (Figure 4A,B), demonstrated comparable or moderately reduced degradation performance with the PUR particle suspension compared to WT SP2. Furthermore, we prepared a nanoparticle suspension of a thermoplastic polyester‐PUR with a chemical composition different from Impranil DLN‐SD (Figure 1C).^[^ ^13a^ ^]^ We determined the release of MDA after the hydrolysis catalyzed by WT SP2, thereby verifying its hydrolytic activity on urethane bonds rather than ester bonds in PUR materials (Figure S5, Supporting Information).

#### 2.2.2 Structural Insights into Activity Variations in UMG‐SP2 Mutants

The crystal structures of UMG‐SP2 solved in this study and their comparison to other related AS family members, especially UMG‐SP1,^[^ ^11^, ^13^ ^]^ now allow us to interpret the results of the mutagenesis studies. When K91 in the catalytic triad was mutated to alanine, the activity was completely lost. S167 near the *cis*S166 has been proposed to play a decisive role in the activity of UMG‐SP2, as verified by an inactive S167A mutant. Intriguingly, K91, which interacts with the other two residues of the catalytic triad, additionally forms a hydrogen bond network with S167 and S185. H215 stabilizes this network through a hydrogen bond to S185 in the substrate‐bound structure. Indeed, replacing H215 with alanine leads to a loss of activity, as does mutation to the negatively charged glutamate, both of which will disrupt this stabilizing effect. Mutating H215 to the positively charged but significantly larger arginine also leads to a loss of activity, likely due to steric hindrance.

**Figure 5:**
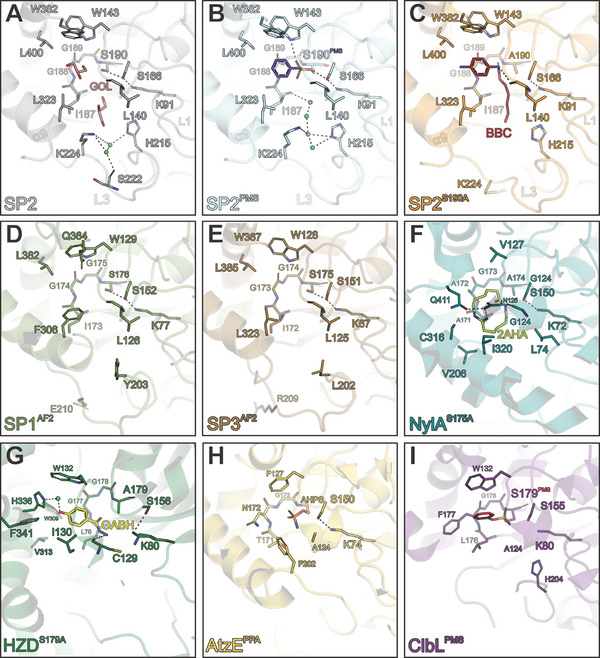
Active site architecture of UMG‐SP2 in comparison to structurally related enzymes of the AS family. Close‐up view of the active site of the A) SP2 structure in grey (glycerol in salmon) and the superimposed B) SP2^PMS^ structure in light blue (phenylmethyl sulfate ester PMS in purple) to the active site of C) SP2^S190A^ structure (light orange) bound to BBC, D) AF2 model of SP1 (SP1^AF2^) in olive, E) AF2 model of SP3 (SP3^AF2^) in brown, F) nylonase A (NylA, PDB‐ID: 3A2P) in forest green bound to a cyclic aminohexanoate dimer (2AHA, light yellow), G) hydrazidase mutant S179A (HZD^S179A^, PDB‐ID: 5H6S) in forest green bound to the substrate 4‐oxidanylbenzohydrazide (OABH, yellow), H) 1‐carboxylbiuret amidase AtzE (PDB‐ID: 6C6G) in light yellow bound to its suicide inhibitor phenyl phosphorodiamidate (PPA, orange), and I) amidase ClbL (PDB‐ID: 8ES6) in purple covalently bound to PMS (red). Relevant secondary structure elements are labeled in (A)–(C). The coloring and molecule representations are the same as in Figure 2.

The primary active site cavity of SP2 accommodates the phenyl and nitrophenyl moieties of the suicide inhibitor PMSF and BBC, respectively, through a set of hydrophobic residues including W143, W382, L400, L319, and L323. The same picture unfolds for nearly all related amidases, except for NylA and partially for AtzE, likely due to their different substrate preference (**Figure** **5**A–I). When L319, L323, or W382 were mutated to alanine, the activity toward various substrates was drastically reduced, likely due to interference with the substrate binding process. Similarly, when V193, which stabilizes W143, was mutated to alanine, only very low hydrolytic activity was observed (Figure 5A,B). Interestingly, W143, L400, and the hydrophobic character of L323 (residue numbering based on UMG‐SP2, Figure 5A–C) are highly conserved among the metagenomic urethanases (Figure 5D,E). A structural equivalent of W143 is also found in HZD and ClbL (Figure 5G,I). The hydrophobic residues L140 and I187 bind to the 4‐hydroxy butyl moiety of the BBC ligand and form the secondary cavity of the active site, which partially accommodates BBC. Mutating either residue to alanine was detrimental to activity (data not shown), as these mutations are likely to interfere with substrate binding. Interestingly, mutations in the neighboring residues of L140 caused distinct effects on the hydrolytic activity of SP2. While G139A demonstrated comparable hydrolysis activity on MDI‐DEG, the A141G variant outperformed WT SP2 significantly in both MDI‐DEG and polyester‐PUR hydrolysis (Figure 4). In the ligand‐free structure, A141 is in the vicinity of a glycerol molecule. At the same time, in the S190A mutant, it contacts the bound BBC substrate, contributing to the hydrophobicity in the substrate pocket. A glycine in place of A141 may have a variety of effects. For example, this mutation may weaken substrate interactions, reducing its release, and improving active site clearance. Alternatively, the increase in backbone flexibility could facilitate stronger substrate binding. However, only more in‐depth structural studies can confirm these hypotheses. The increased activity of the Q399A variant (Figure 4) could be attributed to the same reason as the A141G variant. In addition, a secondary structure element near the active site is destabilized because A399 can no longer contact the backbone amide of G370.

The structure of SP2 bound to BBC also revealed a second substrate binding site flanked by W103, F217, T220, and R325 (Figure 3F). The importance of this site is highlighted by the W103A and R325A mutations, which resulted in lower activity compared to WT SP2 (Figure 4). In the case of W103A, the effect is most likely indirect because W103 contributes to stabilizing the local conformation at the secondary binding site, which is destabilized when mutated to alanine. R325, on the other hand, can directly interact with substrate in the secondary site, through the amphipathic part of the side chain or hydrogen bonds, since the guanidine group of R325 is within 4 Å of BBC's carbamate group, both of which are abrogated by replacing R325 with the smaller alanine. In contrast, T220A and F217A only have a negligible effect on activity. Both residues do not directly interact with the secondary substrate and contribute to the hydrophobic character of the secondary binding site, which is preserved through the change into alanine.

K224, identified to perform a significant conformational change upon substrate binding (Figure 2), is located in the flexible L3 loop (Figure S2A, Supporting Information; Figure 5A–C). This loop can also be found in UMG‐SP1 and UMG‐SP3 (Figure 5D,E), and presumably contributes to a gating mechanism by partially blocking the active site. The A223P/A225P double mutant, which results in a complete loss of activity, highlights the importance of the flexibility of L3. The two flanking prolines lock K224 in place, partially blocking the active site. A similar effect was observed when K224 was mutated into different hydrophobic residues. We speculate that a bulky hydrophobic residue (e.g., W, F, and L) at position 224 would be stabilized through interactions with other hydrophobic residues near the active site (W103, L140, V322, L323), reducing the flexibility of L3 and blocking the binding site. In comparison, when K224 is mutated to the less bulky V or A, only a moderate reduction in activity on MDI‐DEG was observed, rather than a complete loss of activity (Figure 4A,B). A possible explanation is that the flexibility of L3 may not be significantly affected by the hydrophobic interactions resulting from the substitution of K224 with smaller hydrophobic residues. Similarly, when K224 is mutated to G or P, the mutants showed an increase in carbamate hydrolysis activity, again likely due to an increase in L3 flexibility. When K224 was mutated to glutamate, as adopted by SP1 (Figure 5D), activity on MDI‐DEG increased significantly while activity on polyester‐PUR only increased slightly (Figure 4). We explain this effect by a suspension of the gating function of K224, since glutamate likely interacts with H215 directly and the water molecules trapped in the SP2^S190A^ BCC co‐structure are not present. In this scenario, the K224E‐H125 interaction would result in additional interactions with the substrate. Consequently, both substrate binding and product release would occur without a gating mechanism. Mutating D226 to the less bulky alanine enhanced the hydrolytic activity, potentially attributable to the augmented backbone flexibility of L3. This allows K224 to easier switch between pointing toward the active site and the solvent, whereas the D226E mutation does not increase this flexibility. This hypothesis is supported by the observation that the D226E mutant revealed comparable activities on MDI‐DEG as the WT enzyme (Figure 4A,B). Interestingly, the combined mutants K224E/D226A performed only slightly better than the WT enzyme in the hydrolysis of all investigated substrates and lagged behind the individual single precursor variant in MDI‐DEG hydrolysis (Figure 4). This could also be explained by the increased flexibility of L3, which would prevent a more stable K224E‐H215 interaction and counteract its effect as described above.

Among other double variants, only A141G/D226A outperformed the WT enzyme in MDI‐DEG hydrolysis but not in PUR depolymerization (Figure 4). Furthermore, certain mutants, such as K224R and K224T, which exhibited a notable increase or decrease in activity against MDI‐DEG, respectively, cannot be readily elucidated based on our structural data, suggesting that more in‐depth studies are required to gain a better understanding of the multiple mutational effects on UMG‐SP2 catalytic activity.

### 2.3 Molecular Dynamics Simulations of UMG‐SP2

To better understand how the A141G, K224E, D226A, and Q399A mutants can influence the activity of UMG‐SP2, we conducted MD simulations of these variants complexed with MDI‐DEG (Figure 1B). The stable all‐atom and backbone RMSD values (Table S3, Supporting Information) indicate that the overall folding of the WT‐ and the mutant‐SP2 structures were conserved throughout the simulations. The MDI‐DEG remained lodged in each enzyme's active site cavity and adopted an adequate position for a putative hydrolytic reaction with one of its carbonyl groups accommodated in the oxyanion hole and close to the side chain O_γ_ of the catalytic S190 (Table S4, Supporting Information).

To identify the most prevalent conformations of the L3, we merged the trajectories of the 400 ns triplicates ran for the WT‐, K224E‐, and D226A‐SP2:MDI‐DEG complexes and performed a cluster analysis (Table S5 and Figure S6, Supporting Information). When comparing the WT UMG‐SP2 (Figure S7A, Supporting Information) with the K224E variant (Figure S7B, Supporting Information), we observe that the mutation induced a pronounced structural rearrangement of L3. The K224E substitution allowed the establishment of a new ionic interaction between E224 and R325, the latter being in a nearby loop—L4 (residues 325–336, Figure S2A, Supporting Information), which was absent in the WT structure. This local increase in polarity favored the organization of an extensive network of water molecules bridging the two loops (Figure S7B, Supporting Information). Such unique features brought L3 and L4 closer together, as shown by the increased number of contacts and reduced minimum distance between the two regions (Table S6, Supporting Information). The approximation of L3 and L4 created an opening on the enzyme's surface, providing direct access to the active site cavity and exposing both the ligand and surrounding residues to the solvent (Figure S8, Supporting Information). We believe this orifice, absent in the two most populated clusters of the WT complex (cluster 1 in Figure S8A, Supporting Information), may facilitate the diffusion of reaction intermediates/products and the entrance of water molecules required for MDI‐DEG hydrolysis. This could explain the increased enzymatic activity observed in K224E (Figure 4A,B), offering a plausible rationale for its enhanced performance. We thus reason that the MD simulations suggest an interaction between E224 and R325, rather than between E224 and H215; in an intermediate position between the conformations of the substrate‐free and BBC‐bound structures. An additional effect of the K224E mutation is the flexibility increase of L4 (Table S7, Supporting Information), which enabled the loop to move toward the entrance of the active site cavity, resembling a lid that constricts the substrate (Figure S9, Supporting Information). By limiting the conformational freedom of the MDI‐DEG substrate, we believe that L4 can assist in substrate binding and improve MDI‐DEG's orientation within the active site. This is supported by the shorter catalytically relevant distances observed in the K224E mutant versus WT (Table S4, Supporting Information), along with the higher number of contacts established between MDI‐DEG and the enzyme (Table S6, Supporting Information).

Concerning the D226A mutant, the structural comparison between the WT (Figure S10A, Supporting Information) and the D226A variant (Figure S10B, Supporting Information) evidenced noticeable changes in the conformation of L3. The D226A substitution increased L3's flexibility (Table S7, Supporting Information), which seems to make K224 able to explore other regions of the enzyme with its long side chain. In the WT enzyme, K224 does not interact with any specific residue and is mainly stabilized by the solvent (Figure S10A, Supporting Information). In contrast, the D226A variant shows K224 in a completely different region of the enzyme, where it established an ionic interaction with D208 (Figure S10B, Supporting Information). For more than 23% of the total simulation time, the D208(C_γ_)–K224(N_ζ_) distance in the D226A mutant rested below 5 Å, with an average of 8.8 ± 4.5 Å. In the WT structure, only about 1% of the frames exhibited such short distances (average value of 16.0 ± 3.5 Å). As a result, L3 shifted away from L4, as indicated by the increased minimum distance and the reduced number of contacts between the two structures (Table S6, Supporting Information). Although the diverging movement between the two loops exhibits behavior contrary to that observed for the K224E mutant, it seems to yield a similar effect: the formation of a new orifice that enhances the solvent exposure of the active site cavity (Figure S11, Supporting Information), which we believe could be associated with an increased enzymatic activity. Finally, our simulations indicate that the D226A mutant positions the MDI‐DEG more effectively for catalysis, ensuring shorter distances between the substrate's carbonyl group and both the nucleophilic S190 and the enzyme's oxyanion hole (Table S4, Supporting Information). However, the simulations did not completely elucidate the specific reasons behind this phenomenon.

Consistent with our structural analysis, the MD simulations confirm that the D226A mutation increased the flexibility of the L3 backbone. This may enable K224 to form an ionic interaction with D208, which cannot be achieved in the WT enzyme. In this context, the K224–D208 interaction is lost in the K224E mutant, which correlates with the reduced activity of the K224E/D226A double mutant compared to the D226A single mutant. On the other hand, we discovered that the K224E mutation enabled the formation of a new salt bridge between E224 and R325 (in loop L4), which we believe could be beneficial for biocatalysis. However, the increased L3 flexibility caused by the D226A mutation might reduce the frequency of E224‐R325 interactions by affecting how often E224 encounters R325. Consequently, the K224E/D226A double mutant should be less active than the K224E single mutant. Despite this, the E224–R325 interaction should still be observed (albeit less frequently than in the single K224E mutant), which is impossible in the WT enzyme. This may provide insight into why the double mutant retains greater activity than the WT SP2.

The MD simulations offered additional insights into the markedly enhanced depolymerization activity observed in the Q399A mutant discussed above (Figure 4). In the WT enzyme, Q399 is situated within a large hydrophobic pocket formed by 11 residues—W143, V275, L369, W382, L387, I389, F396, A397, A398, L400, and W402—which accommodates the MDA moiety of the MDI‐DEG substrate (Figure S12A, Supporting Information). The Q399A mutation substitutes the large polar glutamine with a smaller nonpolar alanine residue (Figure S12B, Supporting Information). Our simulations revealed that this substitution not only increased local flexibility but also enhanced the hydrophobicity of the active site pocket, thereby not only strengthening interactions with the hydrophobic MDA core of the substrate (Table S6, Supporting Information) but maintaining it in a better position for catalysis (Table S4, Supporting Information).

Finally, our simulations revealed that the A141G mutation results in significantly stronger substrate binding compared to the WT enzyme (Figure S13, Supporting Information). Over 1.2 µs of MD simulations, the A141G mutant formed, on average, 36 additional protein–substrate contacts relative to the WT system (Table S6, Supporting Information). Furthermore, the RMSD of the MDI‐DEG substrate atoms in the A141G mutant was reduced by 0.6 Å versus WT (Table S3, Supporting Information), reflecting enhanced substrate stability. We propose that this increased binding affinity may stem from the elevated flexibility introduced by substituting alanine with glycine, a smaller residue. Notably, the mutation is positioned close to the MDI‐DEG substrate (Figure S13B, Supporting Information), potentially creating additional space within the active site. This augmented flexibility and increased cavity volume may facilitate greater conformational freedom for the adjacent L323 residue, which was found to be crucial for SP2's activity (Figure 4). Consequently, L323 could optimize its hydrophobic interactions, both with the substrate and with neighboring residues L319 and L320 (Figure S13B, Supporting Information), further stabilizing the protein–substrate complex.

## 3 Conclusion

We have successfully solved the crystal structure of the PUR‐hydrolyzing enzyme UMG‐SP2 in a substrate‐free state, as well as in ligand‐bound states with both the inhibitor PMS and the model carbamate substrate BBC (accommodated in inactive SP2 variant S190A) at high resolutions of <2.6 Å. Through structure‐guided engineering, we identified the single variants A141G, D226A, and Q399A, which showed significantly enhanced activity on various (poly)urethane substrates compared to the WT SP2. Notably, Q399A released over 7.4 times more MDA from MDI‐DEG, while A141G showed more than 30.7‐fold improved degradation performance on polyester‐PUR particles based on turbidimetric monitoring. Although double variants combing these beneficial mutations did not further improve but rather impaired UMG‐SP2 activities, the flexible loop L3, containing the mutagenesis hotspots K224 and D226, was recognized as a key structural component for its biocatalysis by MD simulations. Interestingly, while the structures of UMG‐SP1,^[^ ^11^, ^13^ ^]^ UMG‐SP2, and UMG‐SP3^[^ ^11^, ^13^ ^]^ are broadly similar, there are significant differences—especially in the composition of flexible loop regions—that allow template‐specific amino acid substitutions, yielding urethanases with enhanced hydrolytic profiles, that are not necessarily transferable. In summary, the findings in this study advance our understanding of the structure–function relationships of depolymerizing urethanases and greatly highlight their potential to advance PUR recycling within the context of a bio‐based circular plastic economy.^[^ ^4a^ ^]^

## 4 Experimental Section

### Cloning

To obtain the substrate‐free crystal structure, the gene sequence encoding WT UMG‐SP2 (GenBank Accession No. OP972510) with an N‐terminal twin‐Strep tag was codon‐optimized (Proteolutions, Berlin, Germany), synthesized and subcloned into pET28a(+) by GeneScript Biotech (USA). For all other experiments, the gene encoding WT UMG‐SP2 was chemically synthesized by Generay Biotech Co. (Shanghai, China) and subsequently cloned into the pET32a vector.

### Expression and Purification

For the recombinant expression in *E. coli* BL21 (DE3) and purification of SP2, employed for solving the substrate‐free structure, *E. coli* colonies were cultured in 1000 mL lysogeny broth (LB) medium at 37 °C to an optical density at 600 nm (OD_600_) of 0.8 before inducing with 0.5 × 10^−3^ m isopropyl β‐d‐thiogalactopyranoside (IPTG) at 16 °C overnight. Cells were harvested by centrifugation (5000×*g*, 20 min) at 4 °C and resuspended in lysis buffer containing 20 × 10^−3^ m Tris‐HCl (pH 7.5), 200 × 10^−3^ m NaCl, and 1 × 10^−3^ m dithiothreitol (DTT). Cells were disrupted by sonication at 4 °C and the resulting lysate was clarified by centrifugation (20000×*g*, 60 min, 4 °C). The supernatant was added to a column filled with Strep‐Tactin resin in a BIO‐RAD Econo‐Pac chromatography column. The target protein was eluted with lysis buffer containing 10 × 10^−3^ m Desthiobiotin and incubated with tobacco etch virus (TEV) protease overnight to remove the twin‐Strep tag. TEV protease was removed by passing the eluate through a Ni‐NTA agarose column. The target protein was then concentrated using an Amicon Ultra Centrifugal Filter with a molecular cut‐off (MWCO) of 3 kDa and was loaded onto HiLoad 16/600 Superdex 75 pg gel filtration column. Peak fractions were pooled and passed through another Strep‐Tactin column to remove uncleaved target protein. As a final step, the flow‐through was again concentrated to 16 mg mL^−1^ using an Amicon Ultra Centrifugal Filter, 10 kDa MWCO. Protein samples were frozen in liquid nitrogen and stored at −80 °C.

For all other experiments (including the activity assays and solving the ligand‐bound co‐structures of SP2), the pET32a‐UMG‐SP2 plasmid was used to transform *E. coli* BL21(DE3) cells which were grown in LB medium at 37 °C to OD_600_ of ≈0.8 before the induction using 0.4 × 10^−3^ m IPTG at 16 °C for 16 h. Cells were harvested by centrifugation at 5000×*g* for 15 min and then re‐suspended in lysis buffer containing 25 × 10^−3^ m Tris‐HCl (pH 7.5), 150 × 10^−3^ m NaCl, and 20 × 10^−3^ m imidazole, followed by cell disruption with a French Press. Cell debris was removed by centrifugation at 17000×*g* for 1 h. The supernatant was then applied to a Ni‐NTA column with a fast protein liquid chromatography (FPLC) system (GE Healthcare, USA). The target proteins were eluted at an imidazole concentration of ≈100 × 10^−3^ m using a 20–250 × 10^−3^ m imidazole gradient. Each protein was dialyzed against a buffer containing 25 × 10^−3^ m Tris‐HCl (pH 7.5) and 150 × 10^−3^ m NaCl, and subjected to TEV protease digestion overnight to remove the 6 × His tag. The mixture was then passed through another Ni‐NTA column. The untagged protein was eluted with 25 × 10^−3^ m Tris‐HCl (pH 7.5) and 150 × 10^−3^ m NaCl. Then the target proteins were further purified by gel filtration chromatography using a Superdex 200 column to collect a single homogeneous protein elution. The purity of each protein (>95%) was checked by sodium dodecyl sulfate‐polyacrylamide gel electrophoresis (SDS‐PAGE) analysis (Figure S14, Supporting Information). The purified proteins were each concentrated to 10 mg mL^−1^ and stored at −80 °C for crystallization screening. The expression and purification of UMG‐SP2 variants for crystallography and activity assays followed the same procedure as the WT enzyme.

### Site‐Directed Mutagenesis

The plasmids containing genes encoding UMG‐SP2 variants were created using a QuikChange site‐directed mutagenesis kit (Agilent Technologies, Santa Clara, CA) according to the supplier's instructions, with the WT gene as the template. The PCR products were incubated with DpnI (New England Biolabs, Hitchin, UK) to digest the original DNA template before being separately transformed into *E. coli* XL1‐Blue. The mutations were confirmed through sequencing by GENE ray Biotech Co. (Shanghai, China).

### Crystallization, Data Collection, Structure Determination, and Refinement

The substrate‐free WT SP2 was crystallized at 16 mg mL^−1^ in 0.2 m ammonium sulfate, 0.1 m HEPES (pH 7.5), and 35% (w/v) poly(acrylic acid sodium salt) 2100 and crystals appeared after 7 d. For cryo‐protection, crystals were transferred into the precipitant solution containing 20% (v/v) glycerol and then stored in liquid nitrogen. Data were collected on BL14.2 at the BESSY II electron‐storage ring operated by the Helmholtz‐Zentrum Berlin^[^ ^25^ ^]^ and then processed using XDSAPP.^[^ ^26^ ^]^ The structure was solved by molecular replacement with PHASER^[^ ^27^ ^]^ using the previously solved structure of SP2^PMS^ as a replacement model and refined using phenix.refine.^[^ ^28^ ^]^

All other crystallization experiments were conducted at 25 °C using the sitting‐drop vapor‐diffusion method. In general, 1 µL UMG‐SP2 containing solution (25 × 10^−3^ m Tris‐HCl, pH 7.5, 150 × 10^−3^ m NaCl, 0.1 × 10^−3^ m PMSF; 10 mg mL^−1^) was mixed with 1 µL of reservoir solution in 48‐well Cryschem plates and equilibrated against 100 µL of the reservoir solution. The optimized crystallization condition for UMG‐SP2 was 1.26 m (NH_4_)_2_SO_4_, 0.1 m HEPES (pH 7.5). Within 5–6 d, the crystals had grown large enough for X‐ray diffraction. The mutant UMG‐SP2‐S190A crystallized under the same conditions as the WT enzyme. The crystals of UMG‐SP2 and the S190A mutant in complex with 4‐hydroxybutyl (4‐nitrophenyl) carbamate (BBC), were obtained by soaking with mother liquor containing 5 × 10^−3^ m compounds for 1 d. All X‐ray diffraction data sets were collected at the beamline BL17U/BL18U1/BL19U1/10U2 of the National Facility for Protein Science (NFPS) at the Shanghai Synchrotron Radiation Facility. The crystals were mounted in a cryoloop and soaked with cryoprotectant solution (1.26 m (NH_4_)_2_SO_4_, 0.1 m HEPES, pH 7.5, 20% glycerol) before data collection at 100 K. The diffraction images were processed using HKL2000.^[^ ^29^ ^]^ The crystal structure of UMG‐SP2 was solved by the molecular replacement with PHASER program^[^ ^27^ ^]^ from the CCP4 suite^[^ ^30^ ^]^ using the structures predicted by AlphaFold2 (http://biodesign.ac.cn) as the search model. Further refinement was carried out using programs Refmac5^[^ ^31^ ^]^ and Coot.^[^ ^32^ ^]^ Before structural refinements, 5% randomly selected reflections were set aside for calculating Rfree as a monitor.^[^ ^49^ ^]^ Data collection and refinement statistics are summarized in Table S1 (Supporting Information). All figures were prepared by using the PyMOL program (http://pymol.sourceforge.net/).

The numbering of amino acids for SP2 in this study was based on the residue number used for the crystal structures uploaded to the PDB database (Table S1, Supporting Information), which include an N‐terminal expression tag and a linker compared to the sequence deposited in the GeneBank.

### Glycolysis of MDI‐Based Polyether PUR Granules

Commercial polyether‐based PUR (Dongguan Jintian Plastic Materials Co. Ltd., China) was glycolyzed in a round‐bottomed flask equipped with a mixer, thermometer, oil bath heating, and a reflux condenser, as described previously.^[^ ^11^ ^]^ When DEG was used as a glycolysis agent, PUR granules and DEG had a weight ratio of 1:2. The PUR glycolysis reaction was performed at 190 °C for 140 min.^[^ ^33^ ^]^ Next, 10 mL of the resulting glycolysate was added to 1000 mL of water under vigorous stirring, followed by continuous stirring for 2 h and overnight sedimentation. The suspension was centrifuged at 2500×*g* for 1 h, and then the supernatant was discarded. The particles were then resuspended in 100 mL of 0.5% (w/v) SDS, stirred vigorously, and sonicated (Vibra Cell VCX 600 Sonics & Materials Inc.). The particles were then allowed to settle in a cylinder for 1 h before extracting the top 50 mL of the suspension containing the nanosized solid PUR glycolysate particles for further analysis.

### Enzymatic Hydrolysis of MDI‐DEG and the Product Analysis by LC‐MS

1.03 × 10^−6^ m enzyme was incubated in 100 × 10^−3^ m phosphate‐buffered saline (PBS, pH 8.0) containing 1% (w/v) of the nanosized solid PUR glycolysate at 30 °C and stirred at 1000 rpm for 30 min. To stop the reaction, an equal volume of ice‐cold methanol was added and then centrifuged at 12500 rpm for 10 min. The resulting supernatant was used for the analysis by HPLC (Shimadzu LC‐20AD, Japan) equipped with Welch Ultimate XB‐C18 column (5 µm, 4.6 mm × 250 mm). The mobile phase used solvent A (ultrapure water) and solvent B (acetonitrile) to elute the C18 column with 35% acetonitrile, with an elution time of 25 min, a flow rate of 1.0 mL min^−1^, and a wavelength of 254 nm. Mass spectrometry (MS) data were analyzed using the MicroOTOF‐Q II (Bruker, USA) with the electrospray ionization (ESI) and negative ion mode, with a capillary voltage of 4 kV, a gas temperature of 350 °C, a dry gas rate of 5 L min^−1^, Oct 1 RF Vpp = 750 V, and a m/z range of 50–2000. Due to the potential incompleteness of the glycolysis of PUR previously described, a hydrolysis of 1% (w/v) solid glycolysate particles was conducted using an excess of WT SP2 for a 24‐h reaction at 30 °C, estimating a maximum concentration of 1.837 × 10^−3^ m MDI‐DEG present.

### Enzymatic Hydrolysis of Impranil DLN‐SD

For the turbidimetric degradation assay, 200 µg of purified UMG‐SP2 or its variants, 0.4% (v/v) Impranil DLN‐SD (Covestro AG, Germany), and 100 × 10^−3^ m glycine‐NaOH buffer (pH 9.0) were mixed and incubated at 30 °C for up to 21 h under agitation at 200 rpm. OD_400_ was measured to calculate the degradation performance every 30 or 60 min.

### Enzymatic Hydrolysis of Thermoplastic PUR

TPU obtained from Soprema was first used to generate nanoparticles, which were then used as a substrate for enzymatic hydrolysis by WT UMG‐SP2. The monomeric degradation product MDA was quantified using supercritical fluid chromatography (SFC). The procedures for TPU nanoparticle preparation, enzymatic hydrolysis, and SFC analysis were all thoroughly described previously.^[^ ^13a^ ^]^

### Determination of *T*_m_ of UMG‐SP2 and Its Variants

The melting points (*T*_m_) of UMG‐SP2 WT and mutants were determined using DSF on an UNcle system (Unchained Labs, Pleasanton, CA). DSF monitors changes in the intrinsic fluorescence of tryptophan or tyrosine residues during the thermal unfolding of a protein sample without the use of external dyes. The DSF measurements were performed with 1.0 mg mL^−1^ of purified enzymes to determine temperature profiles ranging from 20 to 95 °C at a rate of 0.25 °C min^−1^. The fluorescence data were analyzed using UNcle Analysis software (Unchained Labs, Pleasanton, CA) to calculate the *T*_m_ of a protein, defined as the temperature at which 50% of the protein population transitions from its folded to unfolded state during thermal unfolding. The enzymes appeared to be irreversibly denatured and aggregated after being heated to 95 °C (data now shown), rendering the collection of the refolding ramp from 95 to 20 °C impossible.

### Molecular Dynamics Simulations

To assess the effect of specific point mutations in the overall structure of UMG‐SP2, five distinct models: WT‐, A141G‐, K224E‐, D226A‐, and Q399A‐UMG‐SP2:MDI‐DEG were assembled. These were created using the S190A‐UMG‐SP2:BBC crystal structure as a framework for subsequent modeling. First, all atoms of chain B and all water molecules located at more than 10 Å of any atom of chain A were deleted. Next, the XLEaP module of the AMBER 18 package^[^ ^34^ ^]^ was used to reverse the S190A mutation, transform the co‐crystalized BBC ligand into the MDI‐DEG, and generate the A141G, K224E, D226A, and Q399A mutants. The protonation of all residues at pH 8.0 was set considering the results of the pK_a_ predictor PROPKA software (version 3.5.0).^[^ ^35^ ^]^ The MDI‐DEG was parametrized using the ANTECHAMBER module^[^ ^34^ ^]^ of AMBER 18 (GAFF2),^[^ ^36^ ^]^ and the partial charges were derived by restrained electrostatic potential^[^ ^37^ ^]^ calculations performed at the HF/6‐31G(d) level of theory using Gaussian 09.^[^ ^38^ ^]^ The ANTECHAMBER module was used to add Na^+^ counterions and solvate each UMG‐SP2:MDI‐DEG complex with a rectangular box of TIP3P water molecules within a radius of 12 Å from the surface of the complex.^[^ ^39^ ^]^

The simulations were performed with the GROMACS software (version 2021.5)^[^ ^40^ ^]^ using the AMBER ff14SB^[^ ^41^ ^]^ force field. Each system was submitted to an energy minimization protocol using the steepest descent algorithm.^[^ ^42^ ^]^ The minimized systems were then submitted to MD simulations, in which all bonds involving hydrogen atoms were constrained using the LINCS algorithm,^[^ ^43^ ^]^ allowing for an integration time step of 2 fs. The Verlet cut‐off scheme was used, as well as a non‐bonded cut‐off value of 10 Å. Periodic boundary conditions were considered in all phases, and the Particle Mesh Ewald scheme^[^ ^44^ ^]^ was used to treat non‐bonded Coulombic interactions. The MD simulations started with a 100 ps *NVT* heating phase, where each system was gradually warmed to 30 °C with the V‐rescale thermostat.^[^ ^45^ ^]^ Then, a 2 ns *NPT* equilibration phase was performed to relax the system to 1.0 bar using the Berendsen barostat.^[^ ^46^ ^]^ All solute atoms were kept fixed throughout these two phases. Subsequently, a 2 ns *NPT* equilibration phase was conducted while fixing the geometry of the active site (i.e., K91, S166, I187, G188, and S190) and of a selection of MDI‐DEG atoms. Finally, a 400 ns *NPT* production phase was conducted without any restraints. This MD protocol was performed in three replicates, yielding a total production time of 1.2 µs per complex.

The production trajectories of each set of triplicates were concatenated and submitted to cluster analysis using the Gromos algorithm.^[^ ^47^ ^]^ For the K224E and D226A mutants, a cutoff of 2.1–2.2 Å was applied to the root‐mean‐square deviation (RMSD) of the residues that form the L3. For the A141G and Q399A mutants, a cutoff of 1.35–1.4 Å was applied to the RMSD of the backbone atoms. Tables S5 and S6 (Supporting Information) compile additional data related to the cluster analysis results. The most populated cluster of each UMG‐SP2:MDI‐DEG complex was used for subsequent structural analysis using the Visual Molecular Dynamics (VMD)^[^ ^48^ ^]^ and the PyMOL software. The *gmx mindist* tool from GROMACS was used to calculate the number of contacts between two groups of atoms. The number of contacts was defined as the number of non‐hydrogen atoms from one group that fell within a 6 Å distance of non‐hydrogen atoms from the other group.

## Conflict of Interest

The authors declare no conflict of interest.

## Author Contributions

Z.L., X.H., and L.C. contributed equally to this work. Conceptualization: W.L., R.W., and G.W.; Investigation, formal analysis and validation (protein crystallography): Z.L., X.H., L.C., P.S., W.L., Y.C., D.M.M.J., F.L., G.W., and W.L.; Investigation, formal analysis and validation (mutagenesis, enzyme characterization, and chemical analyses): Z.L., X.H., L.C., Y.B., and L.S.; Investigation, formal analysis and validation (computational simulation): P.P.; Writing – original draft: R.W., G.W., P.P., F.L., Z.L., X.H., and W.L.; Writing – review & editing: all co‐authors; Visualization: G.W., R.W., T.B., P.P., Z.L., X.H., Y.B., and W.L.; Supervision: X.H., G.W., W.L., R.W., U.T.B., P.A.F., M.J.R., U.G., and S.Y.; Project coordination: R.W., G.W., and W.L.; Funding acquisition: W.L., G.W., U.T.B., R.W., P.A.F., and M.J.R.

## Supporting information



Supporting Information

## Data Availability

The data that support the findings of this study are available in the Supporting Information of this article.
