# Supplementary material for: Structure‐Guided Engineering of a Versatile Urethanase Improves Its Polyurethane Depolymerization Activity
Source: Adv Sci (Weinh). 2025 Feb 7;12(13):2416019. doi: 10.1002/advs.202416019 (PMC11967865; doi:10.1002/advs.202416019)
Supplement: Supplementary file 1 — Supporting Information [file ADVS-12-2416019-s001.docx]

Supporting Information

Structure-Guided Engineering of a Versatile Urethanase Improves Its Polyurethane Depolymerization Activity

Zhishuai Li^†^, Xu Han^†^, Lin Cong^†^, Parinita Singh, Pedro Paiva, Yannick Branson, Wenshuo Li, Yangyang Chen, Da’san M. M. Jaradat, Frank Lennartz, Thomas Bayer, Louis Schmidt, Ulrike Garscha, Song You, Pedro Alexandrino Fernandes, Maria João Ramos, Uwe T. Bornscheuer, Gert Weber*, Ren Wei*, and Weidong Liu*

^†^ These authors contributed equally to this work.

**Tables**

**Table S1.** Data collection and refinement statistics of crystals.

|  | SP2 | SP2^PMS^ | SP2^S190A^-BBC |
| --- | --- | --- | --- |
| *Data collection* |  |  |  |
| Space group | *P4_3_2_1_2* | *P4_3_2_1_2* | *P4_3_2_1_2* |
| Unit-cell |  |  |  |
| *a, b, c* [Å] | 88.77, 88.77, 276.44 | 88.43, 88.43, 277.78 | 86.80, 86.80, 276.73 |
| *α* /*β* /*γ* (°) | 90.00/90.00/90.00 | 90.00/90.00/90.00 | 90.00/90.00/90.00 |
| Resolution (Å) | 46.93-2.59 (2.75-2.59) | 50-2.16 (2.22-2.16) | 50-2.40 (2.46-2.40) |
| Unique reflections | 65635 (10605) | 59940 (4311) | 42385 (3050) |
| Redundancy | 14.2 (14.5) | 13.6 (11.4) | 25.1 (25.6) |
| Completeness (%) | 100.0 (100.0) | 99.7 (99.2) | 99.8 (99.1) |
| Average I/σ(I) | 4.7 (0.5) | 12.9 (1.89) | 6.7 (1.25) |
| CC 1/2* | 0.978 (0.311) | 0.99 (0.83) | 0.99 (0.59) |
| *Refinement* |  |  |  |
| R_work_ (95% data) | 0.207 (0.349) | 0.164 (0.245) | 0.206 (0.292) |
| R_free_ (5% data) | 0.235 (0.391) | 0.206 (0.295) | 0.254 (0.321) |
| RMSD bonds (Å) | 0.002 | 0.003 | 0.02 |
| RMSD angles (°) | 0.54 | 0.61 | 0.52 |
| Dihedral angles |  |  |  |
| Most favored (%) | 97.25 | 97.69 | 96.68 |
| Allowed (%) | 2.75 | 2.31 | 3.32 |
| Disallowed (%) | 0.0 | 0.0 | 0.0 |
| no. of non-H atoms / average B [Å^2^] |  |  |  |
| Protein | 6417/48.39 | 6426/39.13 | 6409/46.96 |
| Water | 282/45.88 | 669/48.03 | 384/49.50 |
| Ligand | 65/57.71 | 107/59.79 | 60/54.53 |
| PDB ID code | **9FZW** | **8WDW** | **8XTC** |

a) Values in parentheses are for the outermost resolution shells.

b) CC 1/2* = percentage of correlation between intensities from random half-datasets.^[1]^

**Table S2.** The melting points (Tm) of UMG-SP2 and its variants determined by DSF.

| Enzyme | Tm (°C) |
| --- | --- |
| WT | 44.0 ± 0.1 |
| K91A | 45.2 ± 0.2 |
| G139A | 43.3 ± 0.2 |
| L140A | 42.6 ± 1.0 |
| A141G | 42.6 ± 0.1 |
| S167A | 41.4 ± 0.1 |
| I187A | 45.1 ± 0.1 |
| R218A | 43.5 ± 0.4 |
| K224A | 46.4 ± 0.2 |
| K224E | 43.9 ± 0.2 |
| D226A | 46.4 ± 0.4 |
| L323A | 43.5 ± 0.2 |
| W382A | 40.6 ± 0.3 |
| Q399A | 46.3 ± 0.1 |
| A141G/K224E | 42.1 ± 0.2 |
| A141G/D226A | 45.6 ± 0.2 |
| A141G/Q399A | 43.8 ± 0.3 |
| K224E/D226A | 48.3 ± 0.2 |
| D226A/Q399A | 42.5 ± 0.5 |

**Table S3.** Summary of the RMSD values considering the respective equilibrated structures as references. The values are presented as the average over all frames of the 1.2 μs production simulations (3 replicates × 400 ns) and standard deviations. The locations of loops (L3 and L4) are also depicted in Figure S2. The loop L3 in SP2 is also identical to the loop L3 found in the recently published crystal structure of UMG-SP1.^[2]^

| **Average RMSD (1.2 μs)** | **WT** | **A141G** | **K224E** | **D226A** | **Q399A** |
| --- | --- | --- | --- | --- | --- |
| **Protein / Å** | 2.0 ± 0.2 | 2.3 ± 0.2 | 2.1 ± 0.2 | 2.1 ± 0.3 | 2.2 ± 0.3 |
| **Backbone / Å** | 1.5 ± 0.2 | 1.8 ± 0.2 | 1.7 ± 0.2 | 1.7 ± 0.3 | 1.7 ± 0.3 |
| **L3 (residue 219-226) / Å** | 3.8 ± 1.0 | 4.9 ± 0.8 | 4.4 ± 1.0 | 3.8 ± 1.3 | 4.1 ± 1.1 |
| **L4 (residue 325-336) / Å** | 5.1 ± 1.4 | 7.1 ± 2.0 | 5.6 ± 1.0 | 5.7 ± 1.9 | 6.1 ± 1.9 |
| **MDI-DEG substrate / Å** | 3.6 ± 1.5 | 3.0 ± 0.9 | 3.6 ± 0.9 | 3.5 ± 1.1 | 3.3 ± 1.0 |

**Table S4.** Comparison of three catalytically relevant distances. The values are presented as the average over all frames of the 1.2 μs production simulations (3 replicates × 400 ns) and standard deviations.

| **Average distance (1.2 μs)** | **WT** | **A141G** | **K224E** | **D226A** | **Q399A** |
| --- | --- | --- | --- | --- | --- |
| **MDI-DEG(C_carb_)** – **S190(OG) / Å** | 4.4 ± 1.7 | 3.5 ± 0.4 | 3.8 ± 0.8 | 3.6 ± 0.3 | 3.4 ± 0.3 |
| **MDI-DEG(O_carb_)** – **I187(H) / Å** | 3.4 ± 2.1 | 2.3 ± 0.4 | 2.5 ± 1.2 | 2.2 ± 0.3 | 2.2 ± 0.3 |
| **MDI-DEG(O_carb_)** – **G188(H) / Å** | 3.1 ± 1.7 | 2.1 ± 0.4 | 2.3 ± 1.1 | 2.2 ± 0.5 | 2.0 ± 0.2 |

**Table S5.** Summary of the cluster analysis based on the RMSD of all atoms comprising L3 (residues 219 to 226).

| **UMG-SP2 variant** | **Total frames** | | **RMSD cutoff / Å** | **# of clusters** | **Cluster 1** | **Cluster 2** | **Cluster 3** |
| --- | --- | --- | --- | --- | --- | --- | --- |
| **WT** | | 12000 | 2.20 | 18 | 59.7 %  (7162 frames) | 21.6 %  (2589 frames) | 9.1 %  (1089 frames) |
| **K224E** | |  | 2.10 | 21 | 72.6 %  (8716 frames) | 10.6 %  (1266 frames) | 6.7 %  (805 frames) |
| **D226A** | |  | 2.20 | 20 | 58.2 %  (6984 frames) | 23.8 %  (2856 frames) | 7.4 %  (890 frames) |

**Table S6.** Number of contacts and minimum distance between L3-L4 and between MDI-DEG-Protein. The values are presented as the average over all frames of the 1.2 μs production simulations (3 replicates × 400 ns) and standard deviations.

|  | **L3 (residue 219-226) — L4 (residue 325-336)** | | **MDI-DEG — Protein** |
| --- | --- | --- | --- |
| **UMG-SP2 variant** | **Average number of contacts (1.2 μs)** | **Average minimum distance / Å**  **(1.2 μs)** | **Average number of contacts (1.2 μs)** |
| **WT** | 64 ± 44 | 2.4 ± 0.9 | 750 ± 75 |
| **A141G** | 59 ± 38 | 2.3 ± 0.8 | 785 ± 61 |
| **K224E** | 80 ± 44 | 2.2 ± 0.6 | 764 ± 55 |
| **D226A** | 34 ± 37 | 2.9 ± 1.1 | 747 ± 54 |
| **Q399A** | 46 ± 47 | 2.8 ± 1.1 | 795 ± 58 |

**Table S7.** Summary of the RMSF values calculated for L3 (residues 219 to 226) and L4 (residues 325 to 336). The values are presented as the average over all frames of the 1.2 μs production simulations (3 replicates × 400 ns) and standard deviations.

| **Average RMSF (1.2 μs)** | **WT** | **A141G** | **K224E** | **D226A** | **Q399A** |
| --- | --- | --- | --- | --- | --- |
| **L3 (residue 219-226) / Å** | 1.6 ± 0.5 | 1.6 ± 0.4 | 1.7 ± 0.7 | 2.0 ± 0.7 | 1.8 ± 0.6 |
| **L4 (residue 325-336) / Å** | 1.7 ± 0.3 | 1.9 ± 0.5 | 2.1 ± 0.4 | 1.7 ± 0.2 | 2.3 ± 0.7 |

**Table S8.** Summary of the cluster analysis based on the RMSD of all backbone atoms.

| **UMG-SP2 variant** | **Total frames** | | **RMSD cutoff / Å** | **# of clusters** | **Cluster 1** | **Cluster 2** | **Cluster 3** |
| --- | --- | --- | --- | --- | --- | --- | --- |
| **WT** | | 12000 | 1.40 | 19 | 50.6 %  (6071 frames) | 29.5 %  (3543 frames) | 13.5 %  (1618 frames) |
| **A141G** | |  | 1.35 | 17 | 43.7 %  (5238 frames) | 31.5 %  (3780 frames) | 16.7 %  (2000 frames) |
| **Q399A** | |  | 1.37 | 24 | 50.9 %  (6111 frames) | 13.1 %  (1568 frames) | 9.8 %  (1177 frames) |

**Figures**


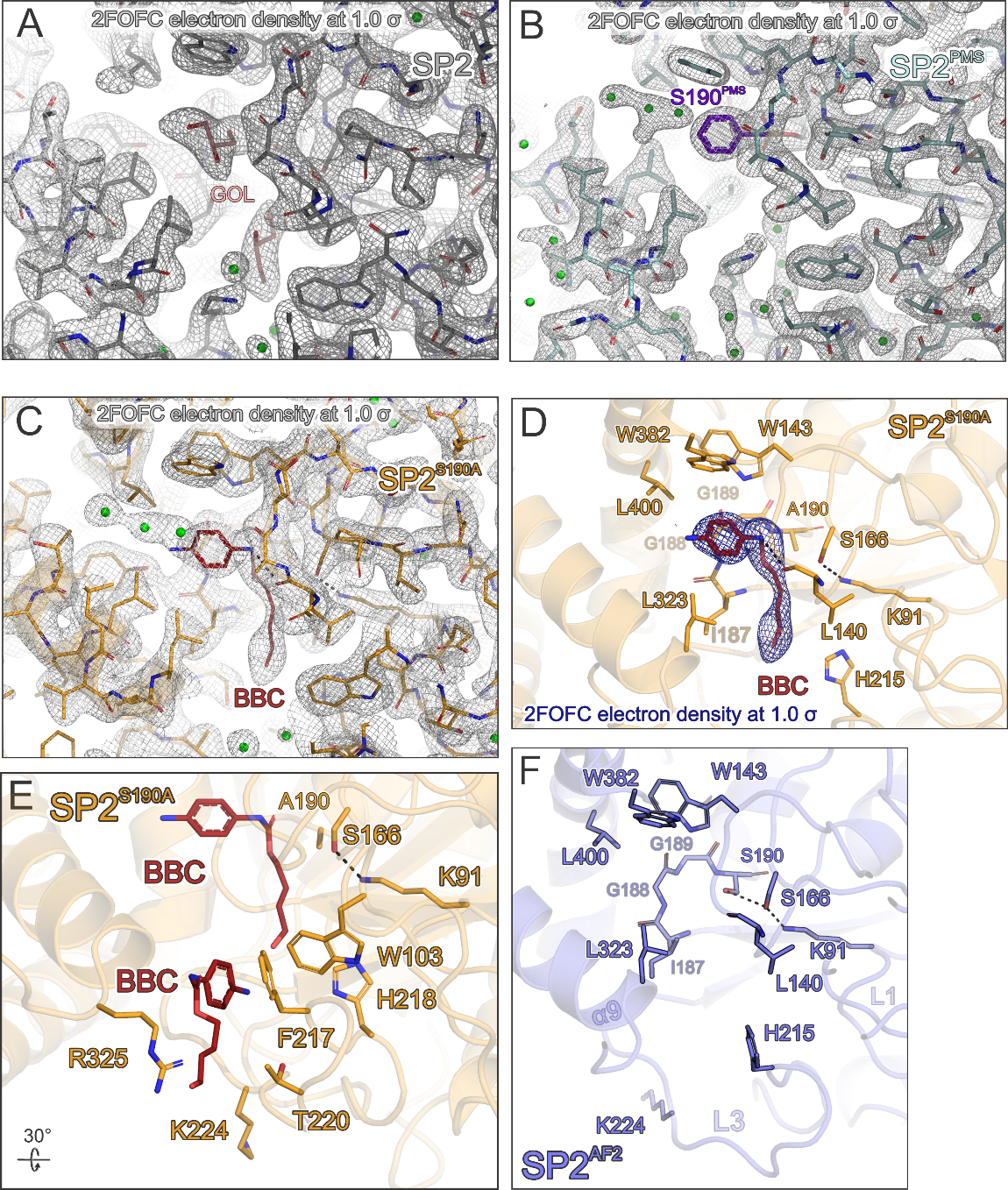


Figure S1. Electron density maps of the SP2 structures, substrate binding modes, and an Alphafold2 model of SP2. (A) The final 2F_o_-F_c_ electron density map (grey mesh) of the substrate-free SP2 structure covering an area around the active site contoured at 1 σ. (B) The final 2F_o_-F_c_ electron density map (grey mesh) of the SP2^PMS^ structure covers an area around the active site, contoured at 1 σ. (C) The final 2F_o_-F_c_ electron density map (grey mesh) covering the active site and the substrate binding cavity of SP2^S190A^, contoured at 1 σ. (D) 2F_o_-F_c_ electron density map covering the substrate BBC. (E) Close-up view of the second molecule of SP2^S190A^ in the asymmetric unit with two BBC substrate molecules bound. (F) Close-up view on the active site of an Alphafold2 model of SP2 (SP2^AF2^) in slate. Colors relate to Figure 2. Water molecules are represented as green spheres.

| **A**  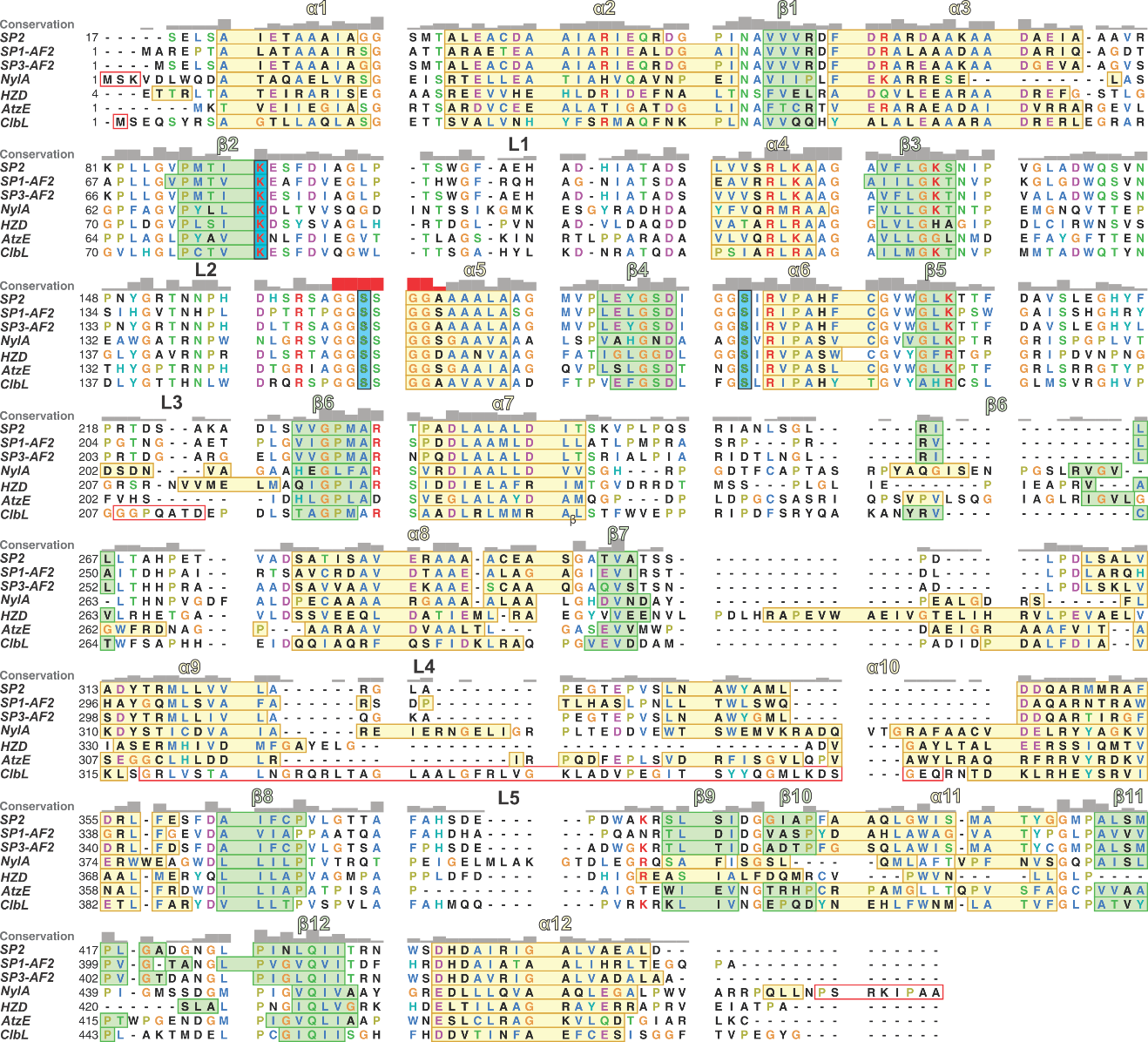 |
| --- |
| **B**  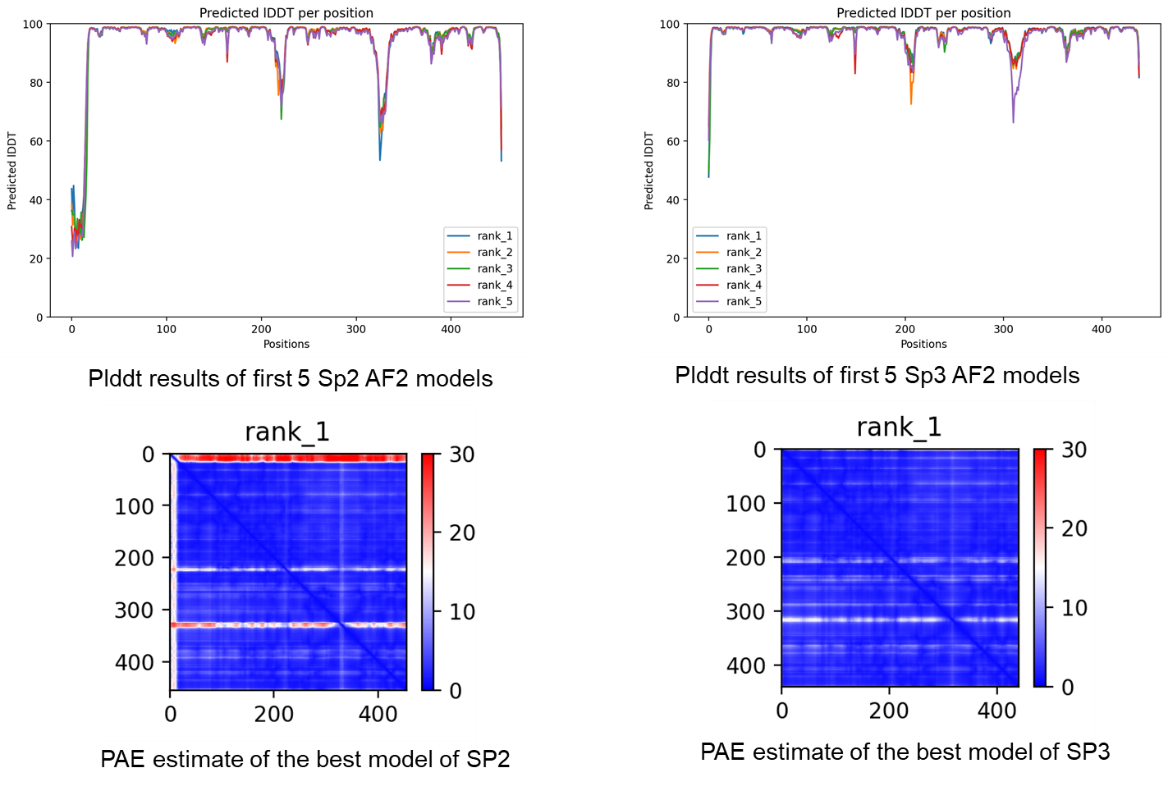 |

Figure S2. (A) Structure-based sequence alignment of UMG-SP2 and related enzymes. The alignment compares SP2 with Alphafold2 models of SP1, SP3 and other structurally characterized enzymes from the amidase (AS) family, i.e., nylonase A (NylA, PDB-ID: 3A2P) hydrazidase (HZD, PDB-ID: 5H6S), 1-carboxybioret amidase (AtzE, PDB-ID: 6C6G) and amidase ClbL (ClbL, PDB-ID: 8ES6) The alignment was prepared by Chimera (https://pubmed.ncbi.nlm.nih.gov/15264254/) employing Clustal Omega (https://www.ebi.ac.uk/jdispatcher/msa/clustalo). Proteins are identified on the left of the aligned sequences with residues numbered. Higher conservation is indicated by grey bars on top of each column, with the AS family motif GGSSGGS shown in red bars. Conserved amino acid residues are colored by chemical properties, basic in blue, acidic in red, hydrophobic in blue, histidine in light blue, glycine in orange, proline in ochre, and glutamine and asparagine in green. Full conservation of the catalytic triad residues is indicated by blue background shading. Secondary structure elements are indicated in the alignment by yellow shading for α-helices and green shading for β-strands and labeled on top of each alignment block, prominent loops are indicated by black letters. Red squares indicate residues missing from the structure or the expression construct. (B) AF2 model quality indication for SP2 and SP3. The AF2-modeled structures are available as separate PDB files.

| **A**  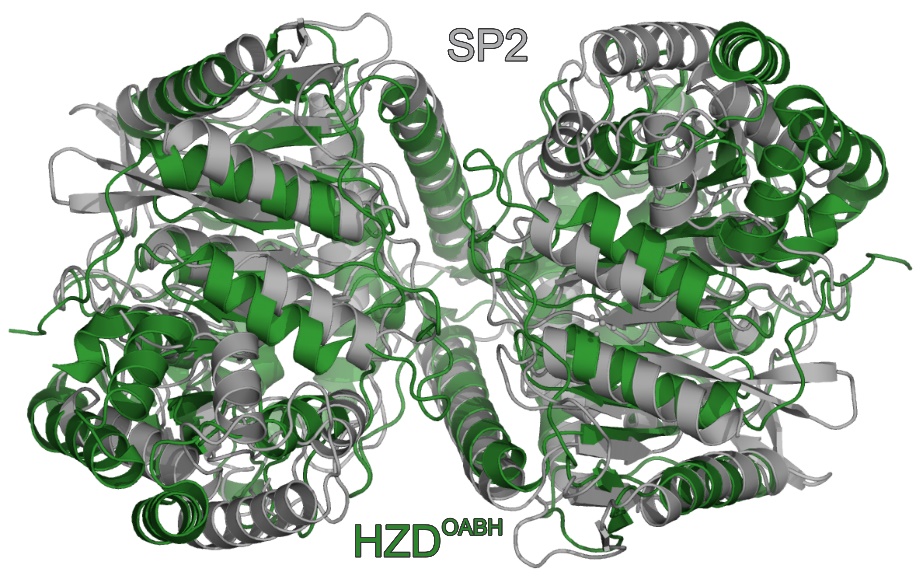 |
| --- |
| **B**  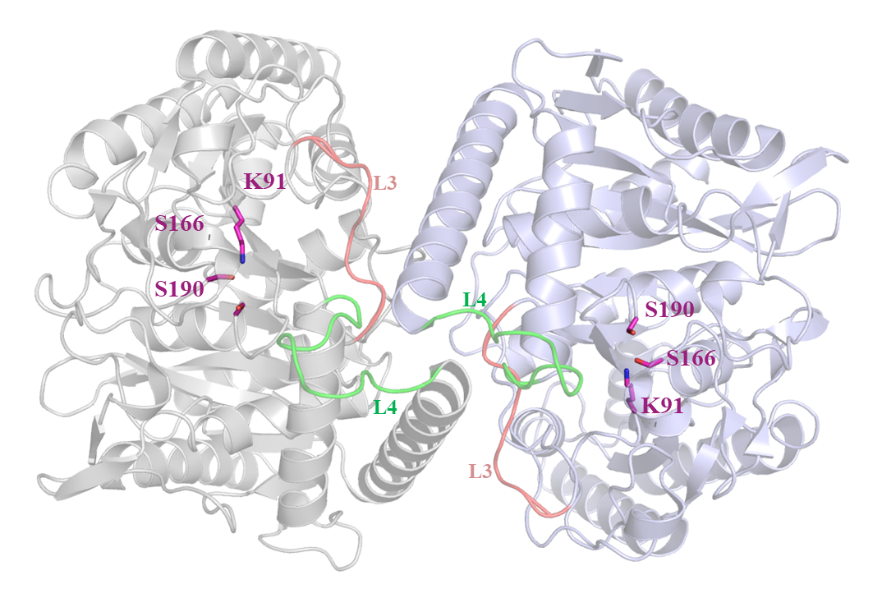 |
| **C**  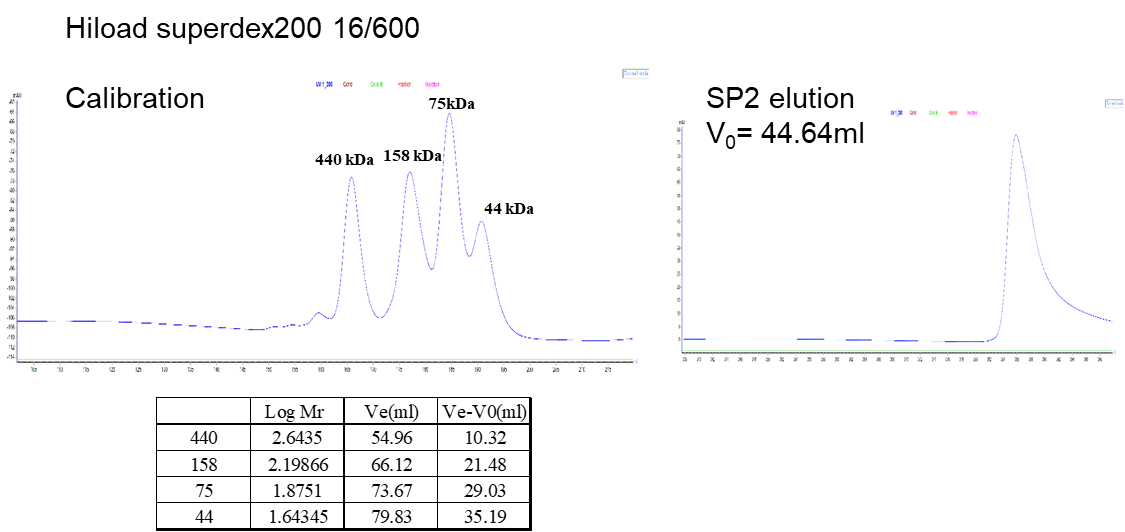 |

Figure S3. (A) Comparison of SP2 and HZD dimerization. The structural superimposition compares the asymmetric unit of SP2 in light blue with hydrazidase (HZD, PDB-ID: 5H6S) in forest green concerning the dimerization mode that may have functional implications. (B) The dimerization of WT SP2. The A chain is grey, the B chain is light blue, L3 is tv-red, L4 is green, and the catalytic triad (K91-S166-S190) is displayed in magenta sticks. (C) The protein elution profile of WT SP2 by size-exclusion chromatography indicates that SP2's functional enzyme is not dimeric.


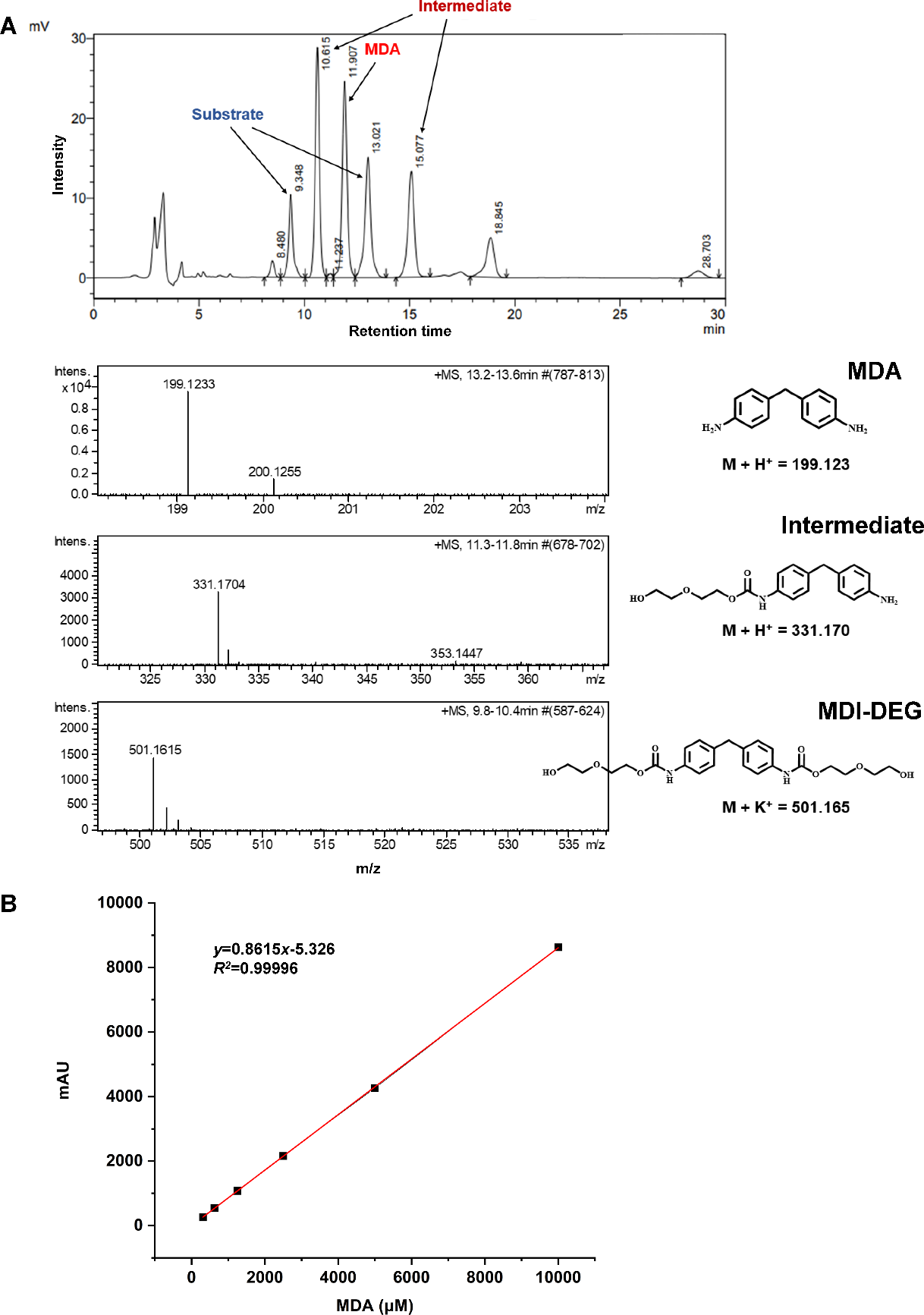


**Figure S4.** (A) LC-MS analysis of MDI-DEG (substrate), the hydrolysis intermediate, and 4,4'-MDA (final hydrolysis product). (B) A calibration curve was obtained using commercially available MDA by plotting the mAU value from the LC-MS analysis against the µM amount of added MDA.

**Figure S5.** A suspension of 0.94 mg/mL of thermoplastic PUR (TPU) nanoparticles (prepared following a protocol published elsewhere;^[2]^ raw TPU was a gift received from Soprema International Ltd.) in a final concentration of 88 mM Tris/HCl buffer (pH 7.5), 88 mM NaCl and 1 mg/mL UMG-SP2 was used for the degradation reactions. Briefly, 200 µL buffer (220 mM Tris/HCl, 220 mM NaCl, pH 7.5) was mixed with 250 µL TPU suspension in water (1.88 mg/mL), and 50 µL of purified UMG-SP2 (10 mg/mL) were added. The reaction was incubated for 24 h at 30°C. Supercritical fluid chromatography (SFC) analysis revealed a formation of 720 nM ± 113 nM MDA. This equals to a product yield of ~0.2% ± 0.03% according to a calculated total yield of ~363 µM of MDA after full hydrolysis. Reactions were performed in triplicates, and the values obtained with the negative control samples (reaction without enzyme) were subtracted.


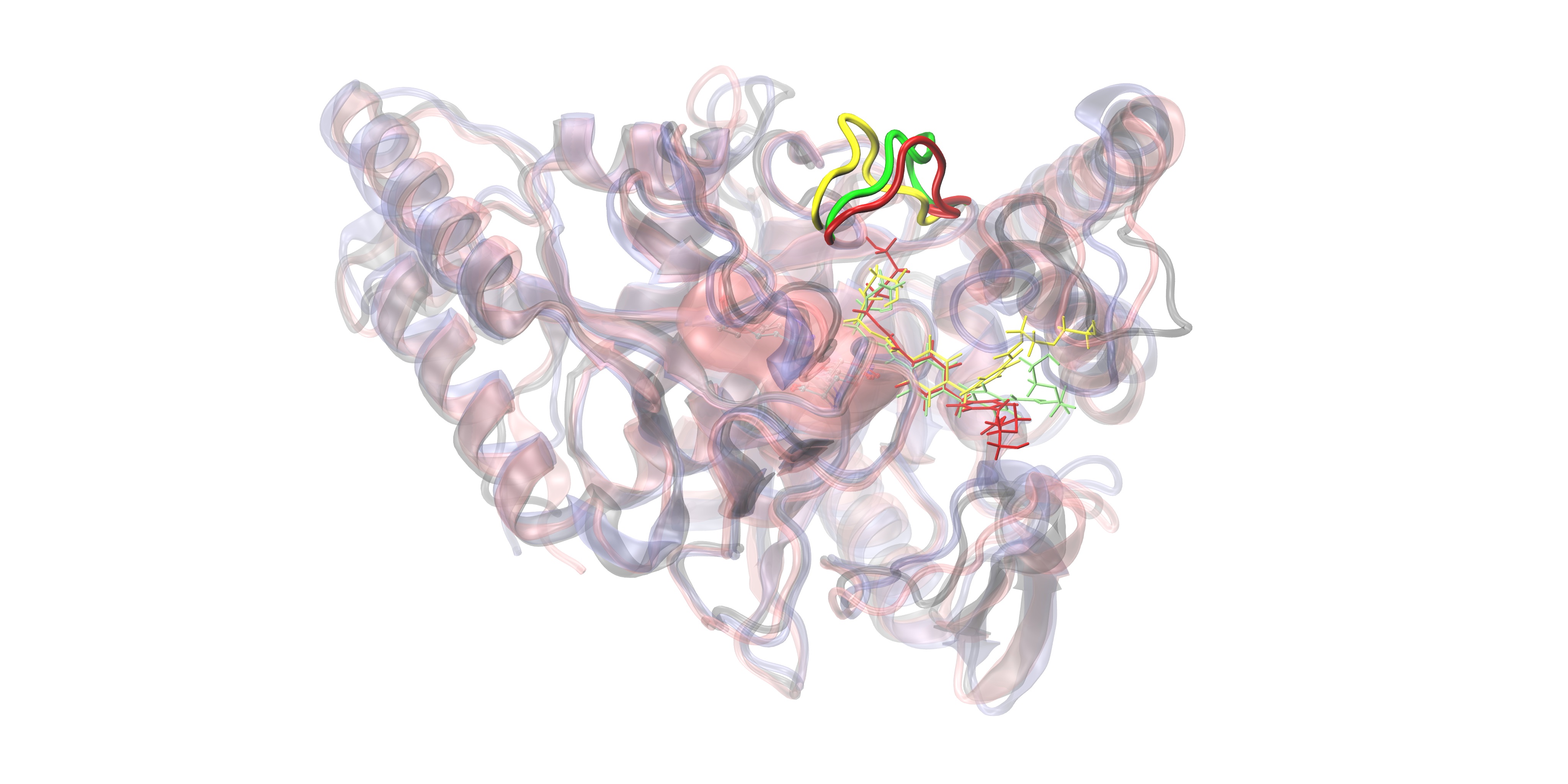


**Figure S6.** 3D structure of the most representative clusters for the WT- (grey), K224E- (ice blue), and D226A-UMG-SP2:MDI-DEG complexes (light pink), aligned by their backbone (shown in cartoon). The loop L3 structure of each cluster is colored according to the following scheme: WT in yellow, K224E in red, and D226A in green. The MDI-DEG is represented as sticks and follows the same coloring scheme. The catalytic triad (K91-S166-S190) is shown both in ball-and-sticks and in light-red translucent surface representations.


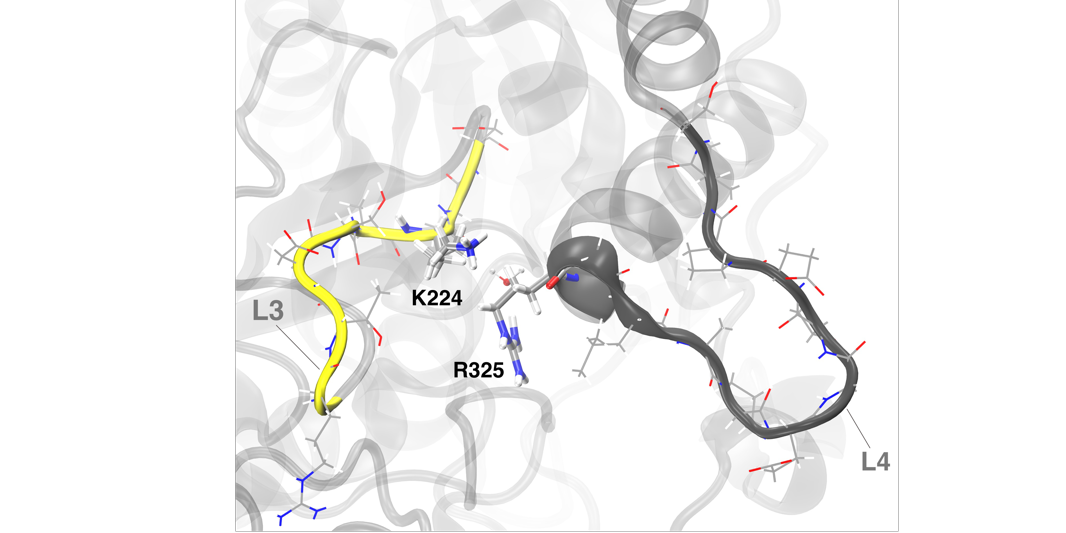
**A B**


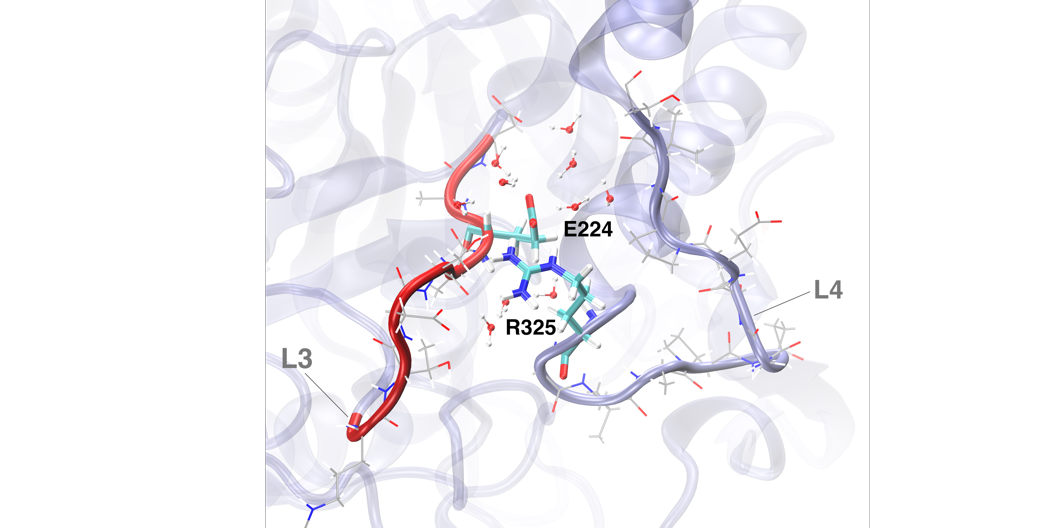


**Figure S7** Close-up of the region surrounding L3 in Cluster 1 of WT (A) versus Cluster 1 of the K224E variant (B). While no specific interactions occur between L3 and L4 in the WT enzyme, a strong ionic interaction between K224 and R325 was observed in the K224E mutant (black dashed lines). It is possible to observe the extensive water molecule network that bridges residues from both loops.

**
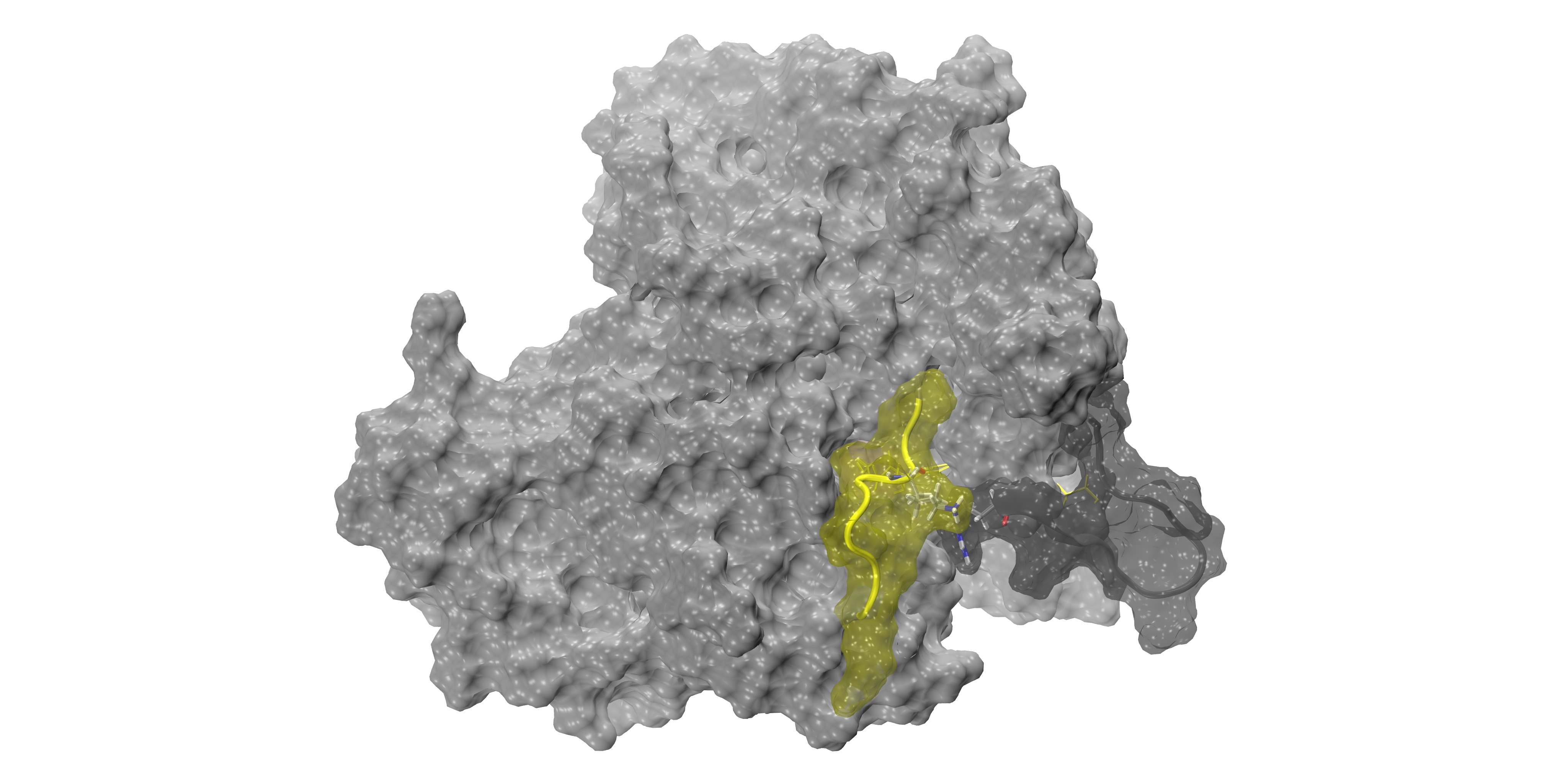
A B**


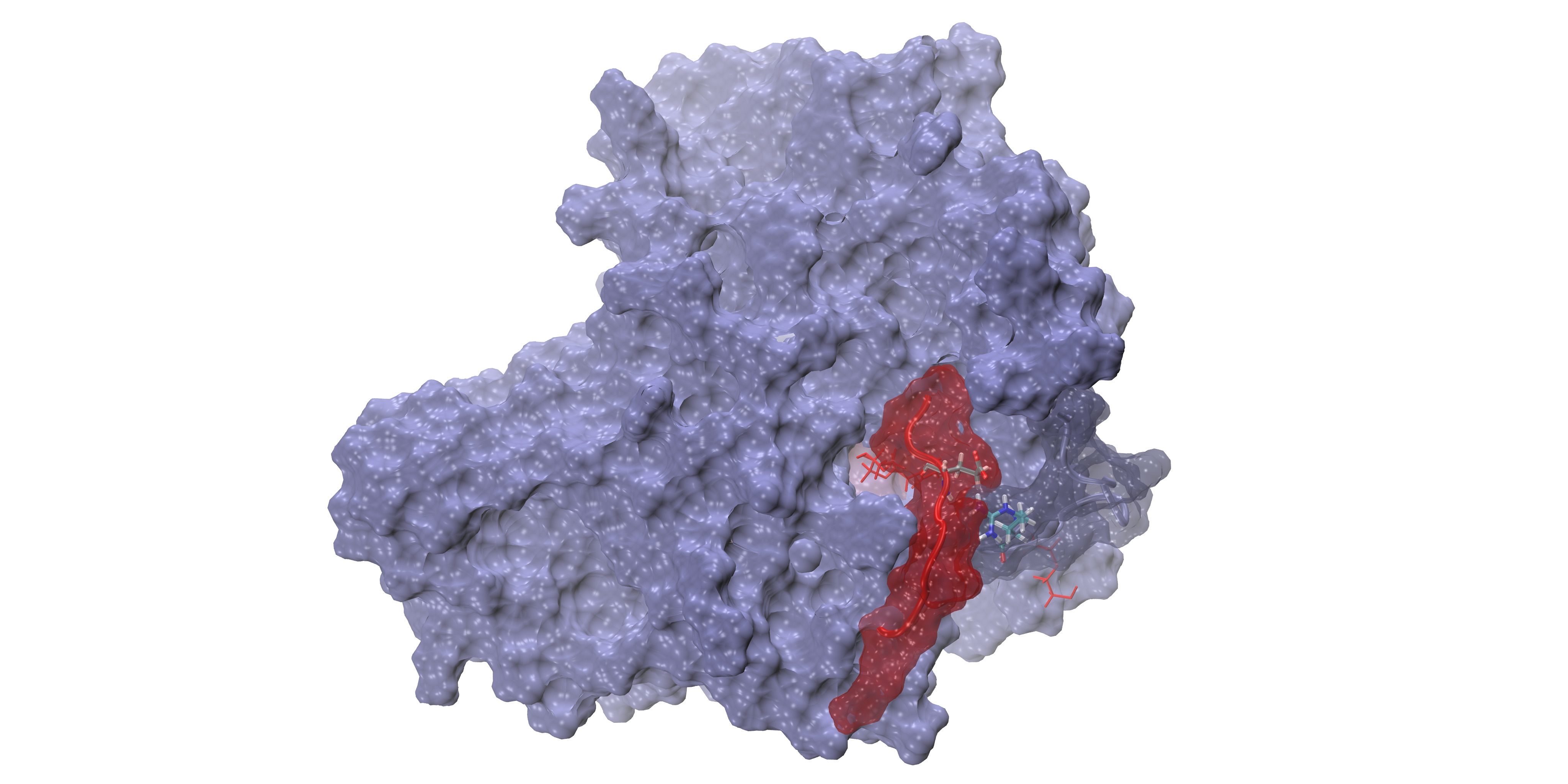


**Figure S8.** Surface representation of the Cluster 1 structures of WT (A) and K224E mutant (B). The approximation of L3 (translucent red surface) and L4 (translucent ice-blue surface) created an opening in the surface of the K224E mutant (orange dashed circle) that leads directly to the active site (right), which does not exist in the WT structure (left).

**A B**


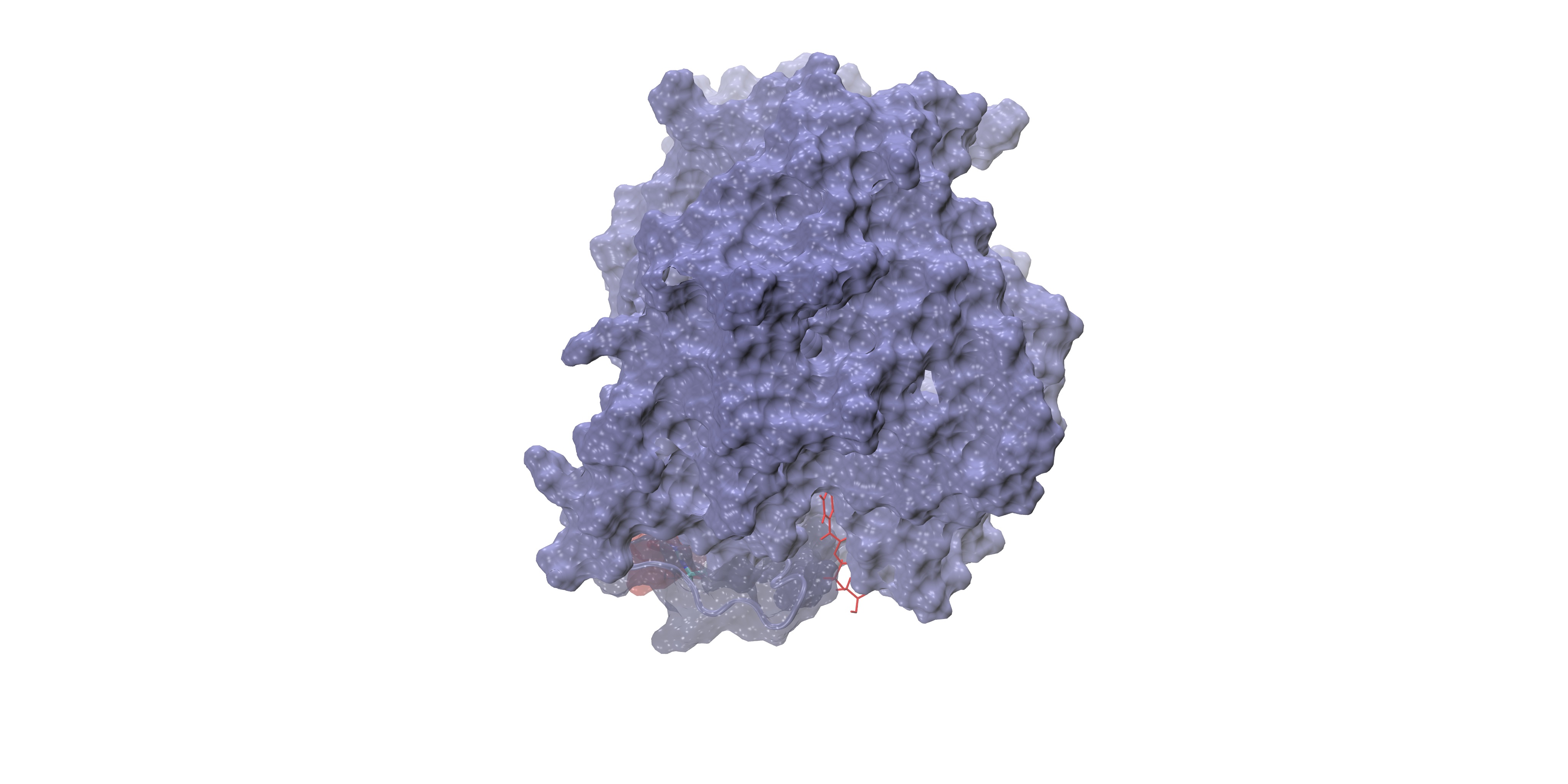


**Figure S9.** Side-view surface representation of the WT (A) and K224E mutant (B) Cluster 1 structures. The red arrows point to the position of L4 in both the WT and K224E variants, which is represented as a grey or ice-blue translucent surface, respectively. The MDI-DEG substrate is shown as sticks and is colored yellow (WT) or red (K224E mutant). The K224E mutation induces a conformational change in L4 which moves toward the entrance of the active site, resembling a lid.


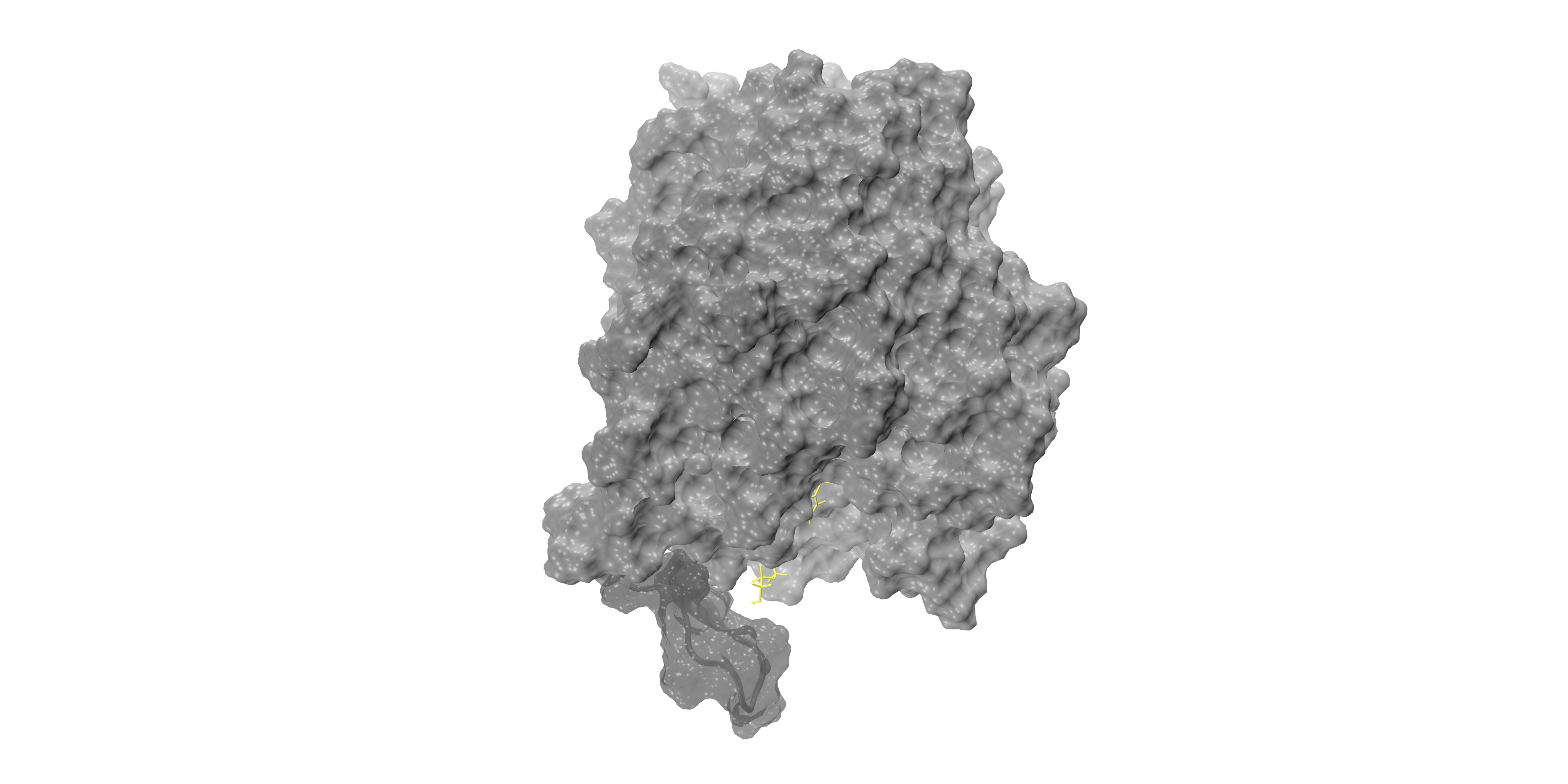


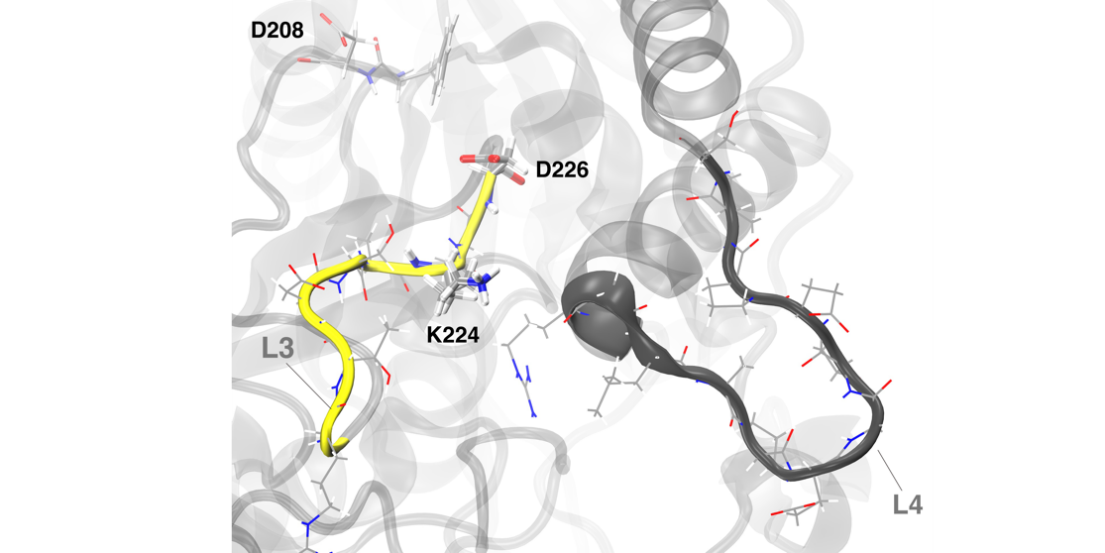
**A B**


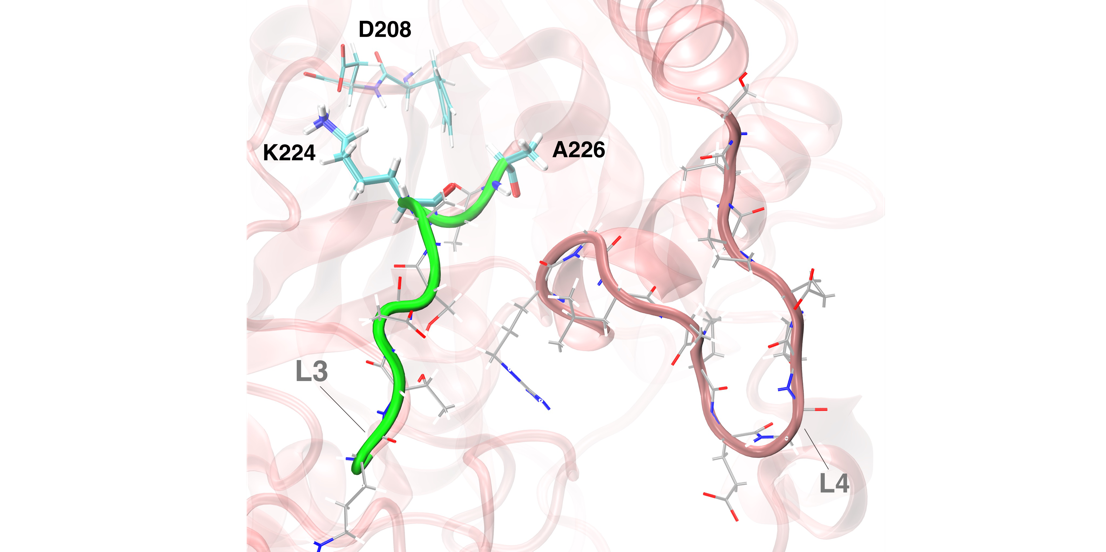


**Figure S10.** Close-up of the region surrounding L3 in Cluster 1 of WT (A) versus Cluster 1 of the D226A variant (B). In Cluster 1 of WT (A), K224 does not interact with any specific residue and is mainly stabilized by the solvent (water molecules not shown for clarity purposes). On the other hand, the Cluster 1 structure of the D226A mutant reveals a large movement of L3, which allowed the positive K224 residue to establish an ionic interaction with D208 (black dashed line).

**A B**
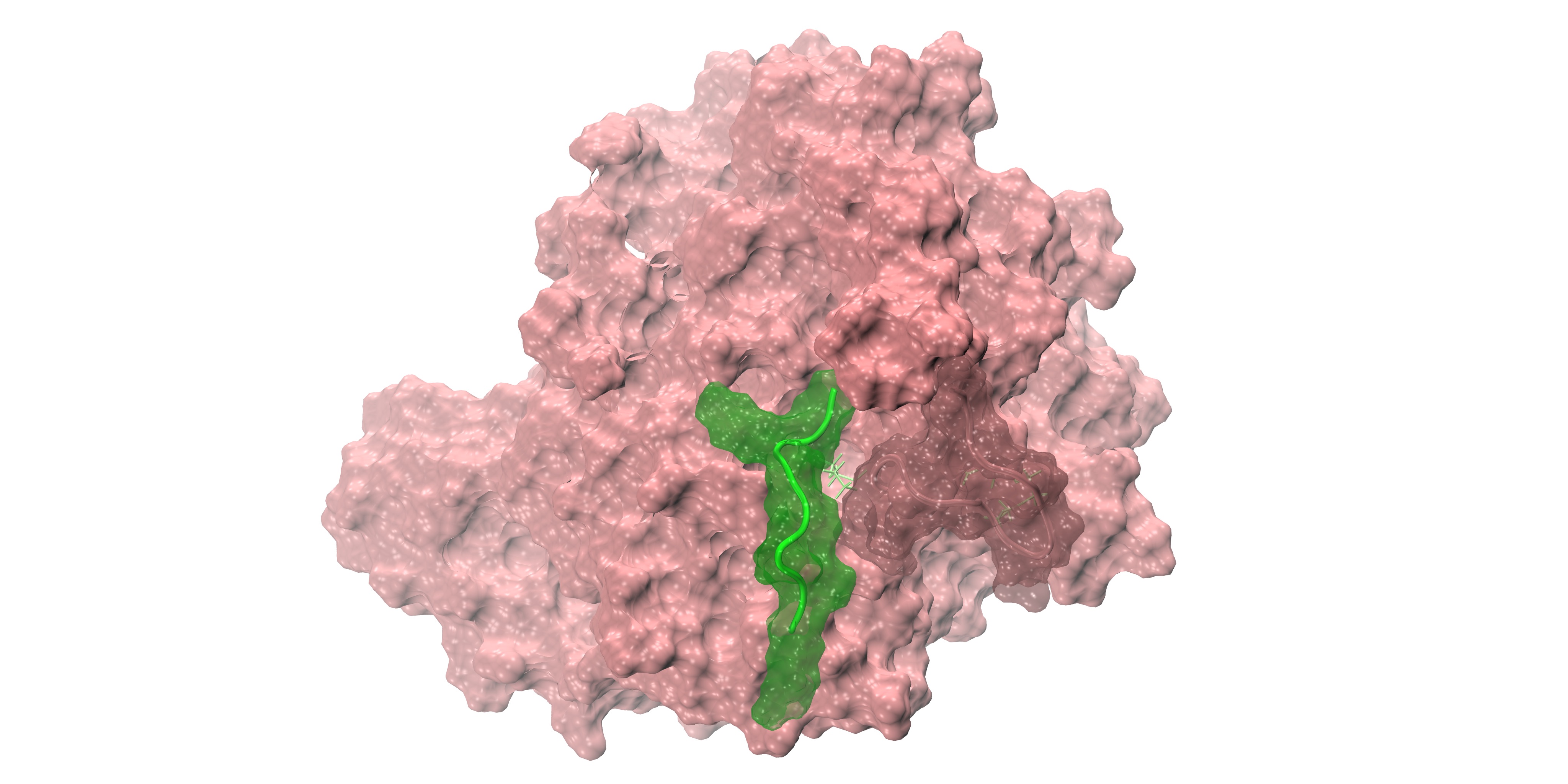

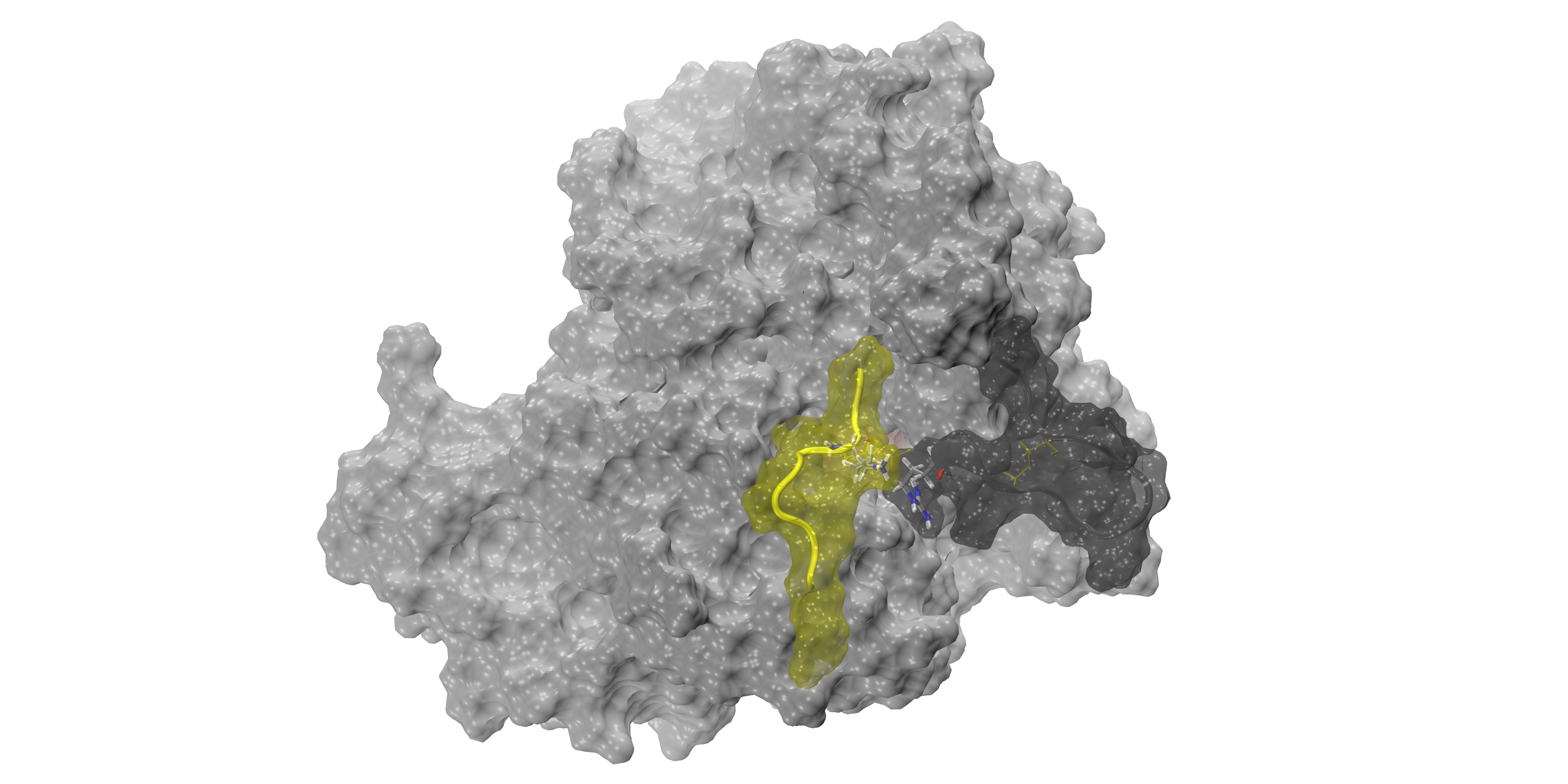


**Figure S11.** Surface representation of the Cluster 1 structures of WT (A) and D226A mutant (B). The diverging movement between L3 (translucent green surface) and L4 (translucent pink surface) created an opening in the surface of the D226A mutant (yellow dashed circle) that leads directly to the active site (right), which does not exist in the WT structure (left).


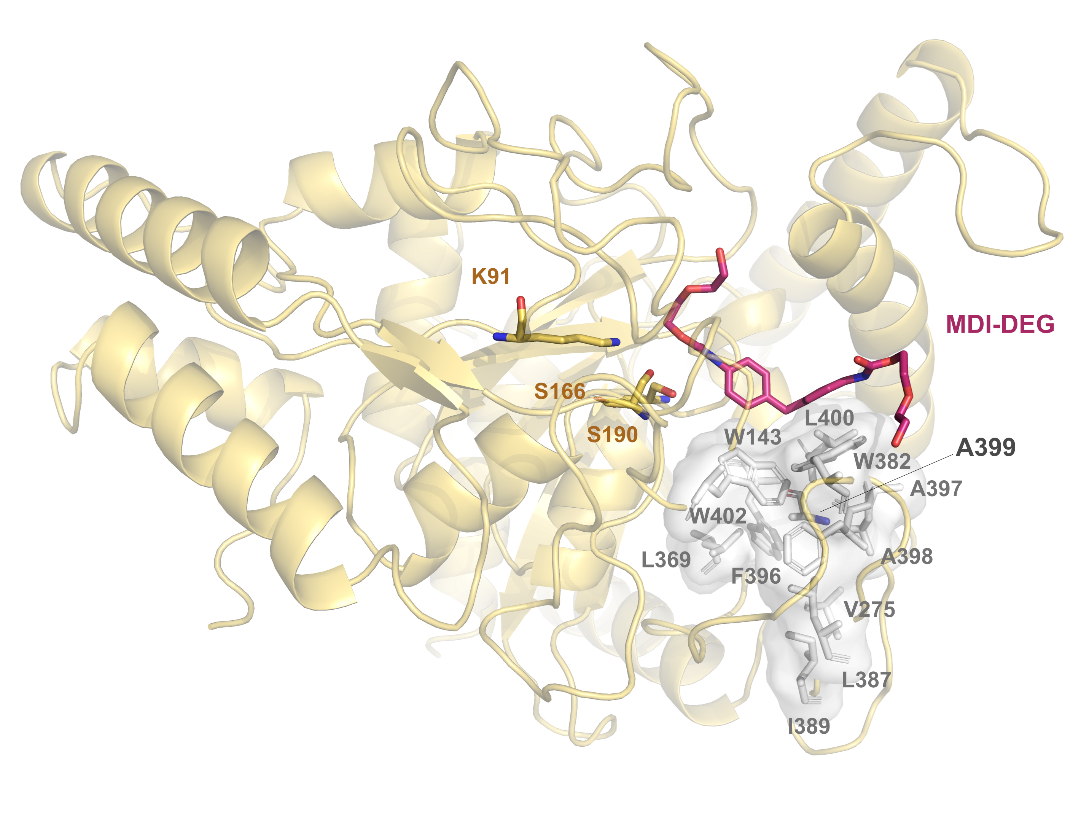

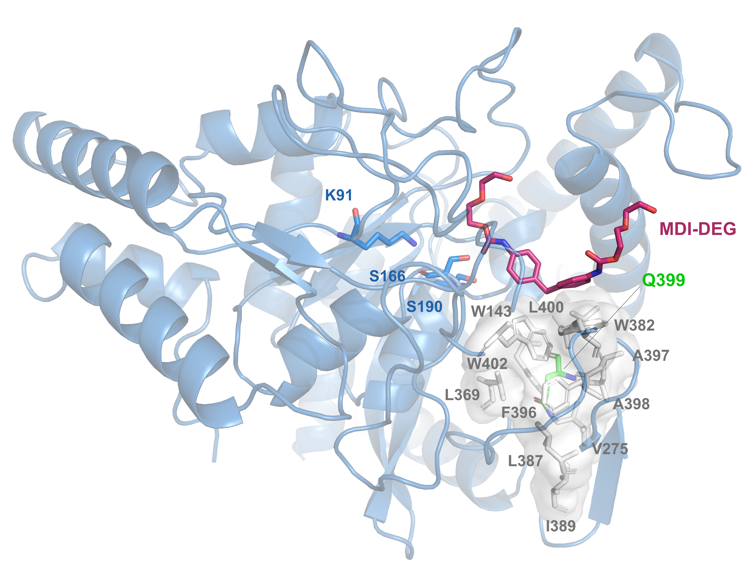
 **A B**

**Figure S12.** Close-up view of the Cluster 1 structures of WT (A) and Q399A mutant (B). In the WT structure (A), the polar glutamine residue at position 399 (Q399, highlighted by the green dashed circle) is located within a predominantly hydrophobic pocket (showed as grey sticks/surface), which accommodates the substrate’s MDA moiety. In the Q399A mutant (B), substituting glutamine with a nonpolar alanine (highlighted by the blue dashed circle) increases the hydrophobicity of this region, strengthening hydrophobic interactions with the substrate, and potentially influencing substrate binding and enzyme activity.


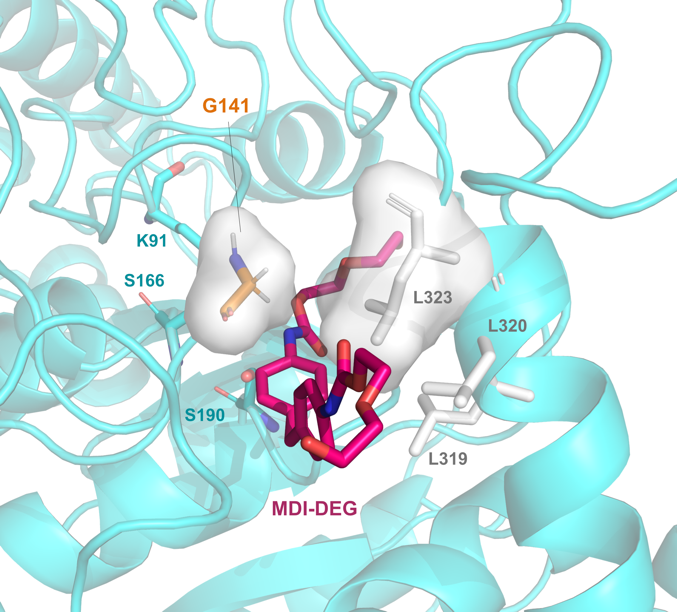

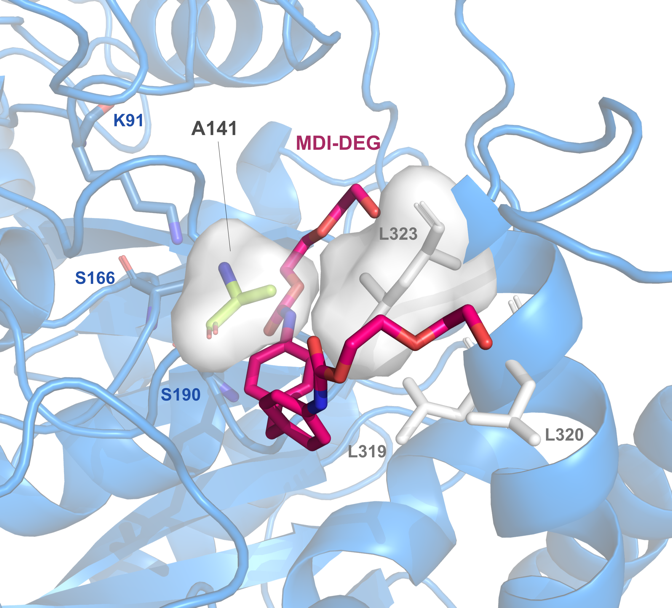
 **A B**

**Figure S13.** Close-up view of the Cluster 1 structures of WT (A) and A141G mutant (B). The alanine-to-glycine substitution in the A141G mutant introduces additional space within the active site cavity (B) and increases the surrounding region's flexibility. This structural change grants L323 greater conformational freedom, enhancing its hydrophobic interactions with the MDI-DEG substrate, as well as with adjacent residues L319 and L320, thereby stabilizing the protein-substrate complex.

| **A**  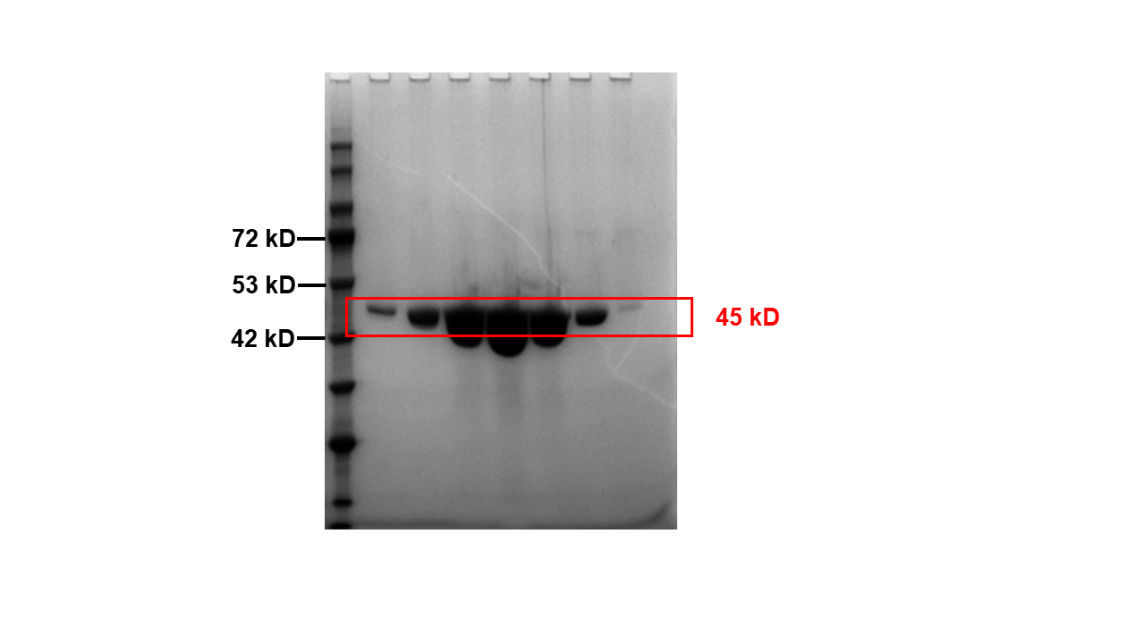 | **B**  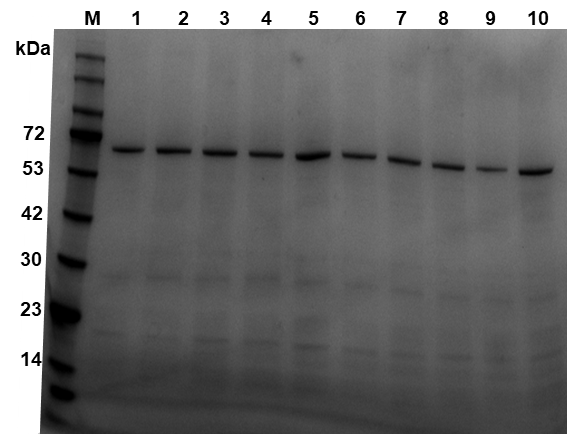 |
| --- | --- |
| **C**  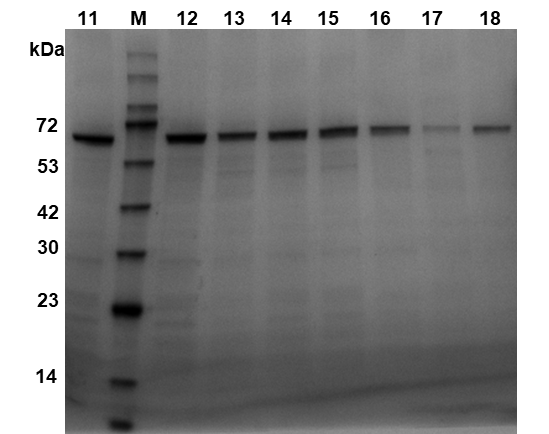 | **D**  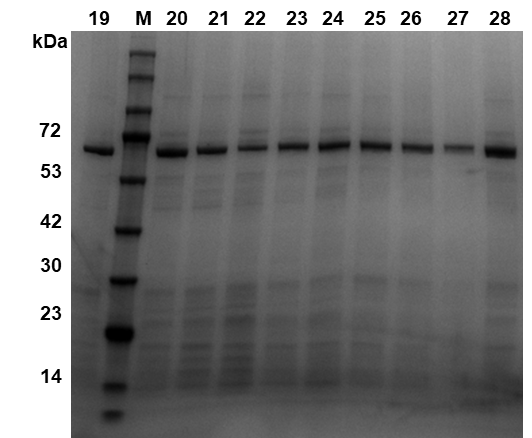 |

**Figure S14.** (A)SDS-PAGE analysis of the purified WT UMG-SP2 (at approximately 45 kDa without any tag, after tandem IMAC and SEC purification steps) used for crystallographic studies. The absence of impurities in the overloaded lanes in the middle of the gel indicates that the purified SP2 sample is highly homogeneous. (B-D) SDS-PAGE analyses of recombinant SP2 and its variants generated (each containing a 16 kDa Trx•Tag™ available in the pET 32a vector used, after IMAC purification) used for enzyme activity characterization in this study: lane M, molecular mass markers; lane 1,WT; lane 2, G139A; lane 3, A141G; lane 4, K224A; lane 5, K224E; lane 6, D226A; lane 7, D226E; lane 8, Q399A; lane 9, T316Y; lane 10, K224E/D226A; lane 11, A141G/K224E; lane 12, V193A; lane 13, L323A; lane 14, W382A; lane 15, A141G/D226A; lane 16, A141G/Q399A; lane 17, D226A/Q399A; lane 18, A223P; lane 19, A225P; lane 20, A223P/A225P; lane 21, F217A; lane 22, T220A; lane 23, W103A; lane 24, R325A; lane 25, K224G; lane 26, K224P; lane 27, K224N; lane 28, K224R.

**References**

[1] P. A. Karplus, K. Diederichs, *Science* **2012**, *336*, 1030-1033.

[2] T. Bayer, P. G.J., L. Berndt, L. Meinert, Y. Branson, L. Schmidt, L. Cziegler, I. Somvilla, C. Zurr, L. G. Graf, U. Janke, C. P. S. Badenhorst, K. S., U. Garscha, M. Delcea, R. Wei, M. Lammers, U. T. Bornscheuer, *Angew. Chem. Int. Ed.* **2024**, *63*, e202404492.
